# Global, regional, and national burden of endocrine, metabolic, blood, and immune disorders from 1990 to 2021, and projections to 2050: a systematic analysis of the global burden of disease study

**DOI:** 10.3389/fendo.2025.1631123

**Published:** 2025-07-25

**Authors:** Jinpai Liang, Hongyan Leng, Xuelian Bai, Linling Li, Tao Qin, Jiazhi Ruan, Guoxing Wang, Wenjuan Zhang

**Affiliations:** ^1^ Department of Endocrinology, Sir Run Run Shaw Hospital, Alaer Hospital, Zhejiang University School of Medical, Alar, Xinjiang, China; ^2^ Department of Endocrinology, Sir Run Run Shaw Hospital, Zhejiang University School of Medical, Hangzhou, Zhejiang, China

**Keywords:** endocrine metabolic blood and immune disorders, global burden of disease, socio-demographic index, age-specific trends, decomposition analysis, future projections

## Abstract

**Background:**

Endocrine, metabolic, blood, and immune disorders (EMBID) are a leading cause of morbidity and mortality worldwide, with substantial regional disparities. Despite advancements in diagnosis and treatment, the burden of EMBID continues to rise. This study aimed to comprehensively assess the global, regional, and national burden of EMBID from 1990 to 2021, with projections to 2050.

**Methods:**

We conducted a systematic analysis using data from the GBD 2021, covering 204 countries and territories, 21 GBD regions, and five Socio-demographic Index (SDI) groups. Age-standardized rates of incidence, prevalence, mortality, and disability-adjusted life years (DALYs) for EMBID were estimated using the GBD analytical framework. Temporal trends were assessed using estimated annual percentage change (EAPC) derived from log-linear regression. Bayesian age-period-cohort (BAPC) models were applied for projections to 2050. Decomposition analysis attributed changes in disease burden to population growth, aging, and epidemiological shifts.

**Results:**

In 2021, the global incidence of EMBID was 79.47 million (95% UI 63.34–98.63 million), with an age-standardized rate of 957.58 (95% UI 766.99–1,183.95) per 100,000, showing a slight decline (EAPC: -0.24% [95% CI -0.35 – -0.12]). Prevalence reached 475.78 million (95% UI 381.23–591.19 million), while deaths rose to 175,902 (95% UI 154,306–190,755; EAPC: 0.75% [95% CI 0.67–0.83]). DALYs totaled 12.86 million (95% UI 9.94–16.98 million), with an age-standardized rate of 157.66 (95% UI 122.38–206.92) per 100,000 (EAPC: -0.09% [95% CI -0.16 – -0.02]). Females had higher incidence and prevalence, while males showed higher mortality. Older adults (≥70 years) experienced the highest burden. Decomposition analysis attributed rising DALYs to population aging (26.02%) and growth (85.83%). Regionally, high-SDI regions showed declining incidence, while low-SDI regions had limited progress. Projections to 2050 indicate declining incidence and prevalence but rising mortality among older adults.

**Conclusion:**

The global burden of EMBID has demonstrated substantial geographical and temporal variability, with lower-SDI regions bearing the highest burden. Addressing these disparities requires enhanced preventive measures, improved healthcare access, and targeted interventions, particularly in low- and middle-income countries.

## Background

EMBID represent a significant component of the global non-communicable disease burden, affecting individuals across all age groups and significantly impacting quality of life and healthcare systems worldwide (1). In recent decades, the prevalence of metabolic disorders such as type 2 diabetes, obesity, and hypertension has surged, especially in rapidly developing regions, reflecting the combined effects of lifestyle changes, aging populations, and increasing exposure to risk factors (2, 3). Despite advancements in medical technology and healthcare delivery, the global burden of EMBID continues to rise, particularly in low- and middle-income countries where healthcare access remains limited (4). Gender and age differences are also evident, with women exhibiting higher prevalence rates for autoimmune diseases, while men demonstrate higher mortality from metabolic conditions (5). Moreover, older adults face disproportionately higher risks of EMBID due to cumulative exposures to risk factors and declining immune function (6).

Socio-demographic factors significantly influence the burden of EMBID, with lower SDI regions experiencing higher prevalence and mortality rates due to limited healthcare resources, poor health education, and unhealthy lifestyles (7, 8). In contrast, high-SDI regions, despite achieving notable progress in healthcare, continue to face rising burdens of EMBID driven by aging populations and lifestyle-related risk factors (9). Effective management of EMBID requires a multifaceted approach, including disease prevention, improved health education, enhanced access to medical services, and targeted interventions tailored to specific populations and regions (10, 11). This study aims to provide a comprehensive analysis of the global and regional burden of EMBID, focusing on the temporal trends, age, sex, and socio-demographic variations from 1990 to 2021 (12). Within the broader EMBID category, this study places particular emphasis on key metabolic and endocrine conditions—namely diabetes mellitus, obesity, metabolic syndrome, and thyroid disorders—due to their high prevalence, modifiable risk profiles, and clinical relevance. Among these, diabetes, obesity, and thyroid dysfunction are the most common and consequential globally, with rising trends driven by urbanization, sedentary lifestyles, and population ageing. These conditions not only shape global EMBID trends but also carry significant implications for clinical practice and public health, underscoring the need for targeted, data-informed strategies. 

## Methods

### Data sources and case definition

This study utilized data from the Global Burden of Disease (GBD) Study 2021, a comprehensive and standardized global database maintained by the Institute for Health Metrics and Evaluation (IHME). The GBD 2021 provides detailed, age-specific, and sex-specific estimates of incidence, prevalence, mortality, and DALYs for diseases, injuries, and risk factors across 204 countries and territories from 1990 to 2021 (13). Data were accessed through the Global Health Data Exchange (GHDx) platform, with rigorous quality control measures to ensure consistency and accuracy (14).

The primary data sources for GBD 2021 included systematic reviews, vital registration data, hospital records, outpatient data, insurance claims, disease registries, and population-based surveys. Data for each condition were synthesized using the Cause of Death Ensemble model (CODEm) for mortality and the Bayesian meta-regression tool DisMod-MR 2.1 for non-fatal outcomes, ensuring robust and consistent estimates. A comprehensive data validation process involving global collaborators and continuous updates by IHME further enhanced data reliability.

EMBID were defined following the GBD 2021 classification system, encompassing four primary categories: endocrine disorders, metabolic disorders, blood disorders, and immune disorders. These categories were further subdivided into specific conditions using the GBD cause hierarchy, mapped to International Classification of Diseases (ICD) codes. This classification ensures consistent diagnosis and comparability across regions and countries.

### Socio-demographic index

The SDI is a composite measure used in the GBD framework to account for variations in development and health outcomes across regions (14). SDI is calculated as the geometric mean of three key indicators: per capita income, average educational attainment in the population aged 15 years and older, and total fertility rate among women under 25 years. Based on SDI values, countries and territories were categorized into five groups: low, low-middle, middle, high-middle, and high SDI regions. This classification allowed for stratified analysis of EMBID burden and trends by SDI level.

### Age-Period-Cohort model

To assess the temporal dynamics of EMBID, we employed an Age–Period–Cohort (APC) analysis, which decomposes the disease burden into three fundamental components (15):

Age Effect (*α*): Reflects age-specific differences in disease risk, capturing the impact of biological and demographic factors.

Period Effect (β): Represents temporal changes that affect all age groups simultaneously, accounting for shifts in healthcare, public awareness, and diagnostic practices.

Cohort Effect (γ): Captures generational differences, indicating how exposure to risk factors varies among birth cohorts.

The APC model is mathematically expressed as:


$$Y_{ijk}=\mu +\alpha_{i}+\beta_{j}+\gamma_{k}+\epsilon_{ijk}$$

where $Y_{ijk}$ represents the observed disease burden for age group *i*, period *j*, and cohort *k*; is the overall intercept; and $\epsilon_{ijk}$ is the random error term.

### Bayesian Age-Period-Cohort model

To project future disease burden, we applied the Bayesian Age–Period–Cohort (BAPC) model, which extends the traditional APC framework by incorporating Bayesian inference via Markov Chain Monte Carlo (MCMC) simulation. Convergence of MCMC chains was assessed using the Gelman–Rubin diagnostic. The selection of the BAPC model was based on three primary considerations. First, the model has demonstrated methodological robustness and flexibility in prior GBD research. Second, it supports probabilistic forecasting and generates stable estimates across time and geography. Third, it ensures methodological alignment with the GBD framework, allowing for cross-national and temporal comparability.

Although customized models may offer higher precision for individual conditions, the use of BAPC provides consistency and scalability essential for multicountry, multi-disease analyses. EMBID encompasses diverse disorders with varying etiologies and determinants, and while grouped for analytic consistency, such aggregation may limit disease-specific inference.

Finally, although the BAPC model may yield narrow uncertainty intervals under dense, stable time series—as is the case in GBD data—these reflect internal model certainty rather than the full spectrum of real-world variability, and should be interpreted accordingly. Future research may explore disease-specific or hybrid modeling approaches to enhance precision where appropriate.

### Decomposition analysis

Decomposition analysis was used to attribute changes in burden to three factors: population growth, population aging, and epidemiological shifts. The method involves:


△Y=△P+△A+△E

where △Y is the total change in burden. △P is the change attributable to population growth, calculated as the difference between the expected and actual burden if population size remained constant. △A is the change due to population aging, calculated by holding age-specific rates constant while allowing age distribution to change.  △E is the change driven by changes in age-specific rates, calculated as the change in age-specific rates while holding population structure constant.

### Statistical analysis

Age-standardized rate per 100,000 people were computed using the formula:


$$\;Age-standardized\;rate=\frac{\sum_{i=1}^{N}a_{i}w_{i}}{\sum_{i=1}^{N}w_{i}}$$

where $a_{i}$ is the age-specific rate in the i-th age group, and $w_{i}$ represents the number of people in the same age group among the GBD standard population, and N is the number of age groups (15).

The EAPC in age-standardized rate was computed to evaluate trends over time (16) This involved fitting a linear regression model to the natural logarithm of the age-standardized rate:


ln(age−standardized rate)=α+β×year+ϵ

where α and β are the intercept and slope, respectively, and ϵ is the error term.

The EAPC was then calculated as:


$$EAPC=100\times (e^{\beta }-1)$$

with 95% CIs estimated from the regression model.

A trend was considered statistically significant if the 95% CI of the EAPC did not include zero (17). Smoothing spline models were used to evaluate the relationship between EMBID burden and the SDI across the 21 regions and 204 countries and territories. Spearman correlation analysis was employed to estimate the association of age-standardized rate with SDI, with p<0.05 indicating statistical significance.

Analyses and graphical representations were executed using the R statistical software (version 4.2.0). A two-tailed P-value below 0.05 was deemed statistically significant.

## Results

### Overall global burden trends (1990–2021)

From 1990 to 2021, the global burden of EMBID exhibited distinct trends across incidence, prevalence, mortality, and DALYs. Globally, incident cases rose from 49.09 million (95% UI: 39.52–61.10 million) to 79.47 million (95% UI: 63.34–98.63 million)(**Supplementary Table S1**), while the age-standardized incidence rate (ASIR) declined from 1,002.59 (95% UI: 804.69–1,240.50) to 957.58 (95% UI: 766.99–1,183.95) per 100,000 population (EAPC: -0.24% [95% CI: -0.35 – -0.12]). Prevalent cases increased from 284.95 million (95% UI: 224.22–354.87 million) to 475.78 million (95% UI: 381.23–591.19 million), with the age-standardized prevalence rate (ASPR) declining from 5,875.42 (95% UI: 4,697.49–7,329.00) to 5,690.89 (95% UI: 4,547.25–7,102.71) per 100,000 (EAPC: -0.17% [95% CI: -0.33 – -0.02]). Deaths more than doubled, rising from 78,445 (95% UI: 67,529–89,083) to 175,902 (95% UI: 154,306–190,755), and the global age-standardized death rate (ASDR) increased from 1.87 (95% UI: 1.65–2.09) to 2.17 (95% UI: 1.90–2.36) per 100,000 (EAPC: 0.75% [95% CI: 0.67–0.83%])(**Table 1**). Notably, the increase in ASDR was more pronounced among males (EAPC: 1.00% [95% CI: 0.91–1.10%]) than females (EAPC: 0.52% [95% CI: 0.43–0.61%]). Total DALYs rose from 8.18 million (95% UI: 6.16–10.87 million) to 12.86 million (95% UI: 9.94–16.98 million), although the age-standardized DALY rate showed a slight decline, from 164.34 (95% UI: 124.28–219.40) to 157.66 (95% UI: 122.38–206.92) per 100,000 (EAPC: -0.09% [95% CI: -0.16 – -0.02]).

**Table 1:** Global and regional age-standardized deaths, DALYs, incidence, and prevalence rates of EMBID by sex, age, and SDI regions, 1990–2021 (EAPC).

	ASMR,95%UI		EAPC,1990-2021	ASDR,95%UI		EAPC,1990-2021	ASIR,95%UI		EAPC,1990-2021	ASPR,95%UI		EAPC,1990-2021
	1990	2021		1990	2021		1990	2021		1990	2021	
Global	1.87 (1.65, 2.09)	2.17 (1.90, 2.36)	0.75 (0.67, 0.83)	164.34 (124.28, 219.40)	157.66 (122.38, 206.92)	-0.09 (-0.16, -0.02)	1002.59 (804.69, 1240.50)	957.58 (766.99, 1183.95)	-0.24 (-0.35, -0.12)	5875.42 (4697.49, 7329.00)	5690.89 (4547.25, 7102.71)	-0.17 (-0.33, -0.02)
Sex												
Male	1.85 (1.64, 2.00)	2.32 (2.03, 2.50)	1.00 (0.91, 1.10)	128.06 (103.62, 162.28)	127.48 (102.94, 157.51)	0.09 (0.04, 0.14)	650.80 (517.36, 812.49)	619.24 (492.28, 773.08)	-0.20 (-0.31, -0.10)	3772.87 (2972.23, 4757.24)	3660.74 (2903.58, 4598.10)	-0.10 (-0.23, 0.03)
Female	1.89 (1.54, 2.26)	2.04 (1.71, 2.28)	0.52 (0.43, 0.61)	199.99 (142.60, 275.33)	187.60 (139.53, 257.12)	-0.21 (-0.30, -0.11)	1345.90 (1078.03, 1659.04)	1289.22 (1032.28, 1592.15)	-0.25 (-0.38, -0.12)	7940.72 (6371.35, 9859.46)	7685.72 (6157.72, 9529.15)	-0.21 (-0.38, -0.04)
SDI												
High SDI	2.69 (2.56, 2.78)	3.70 (3.41, 3.87)	1.38 (1.24, 1.53)	162.51 (136.62, 198.42)	182.97 (156.56, 219.99)	0.54 (0.42, 0.67)	658.23 (533.90, 814.61)	706.46 (573.40, 873.05)	0.17 (-0.02, 0.36)	4266.04 (3413.45, 5288.06)	4556.39 (3666.10, 5617.05)	0.12 (-0.14, 0.38)
High-middle SDI	1.61 (1.45, 1.99)	1.58 (1.39, 1.79)	0.15 (-0.03, 0.32)	173.91 (130.31, 236.74)	161.64 (120.77, 220.76)	-0.19 (-0.26, -0.12)	1079.48 (865.36, 1333.51)	1100.70 (878.60, 1359.39)	0.04 (-0.06, 0.15)	6565.33 (5229.35, 8217.46)	6878.55 (5411.26, 8645.39)	0.19 (0.05, 0.33)
Middle SDI	1.83 (1.46, 2.14)	1.96 (1.56, 2.19)	0.42 (0.33, 0.51)	189.33 (138.10, 256.10)	169.06 (128.56, 228.37)	-0.34 (-0.41, -0.26)	1254.56 (1003.24, 1548.90)	1132.75 (907.03, 1399.86)	-0.38 (-0.48, -0.28)	7300.14 (5865.82, 9138.59)	6695.15 (5365.49, 8342.25)	-0.31 (-0.44, -0.17)
Low-middle SDI	1.05 (0.83, 1.30)	1.31 (1.06, 1.53)	0.96 (0.89, 1.03)	129.54 (93.50, 177.07)	127.11 (95.11, 169.17)	-0.10 (-0.18, -0.01)	932.87 (743.79, 1152.19)	836.19 (668.47, 1032.81)	-0.54 (-0.67, -0.42)	5041.99 (4040.70, 6308.38)	4638.11 (3718.33, 5775.07)	-0.48 (-0.62, -0.34)
Low SDI	1.61 (1.09, 2.17)	1.53 (1.09, 1.95)	-0.21 (-0.28, -0.14)	142.15 (99.76, 193.96)	130.46 (95.23, 170.70)	-0.41 (-0.50, -0.33)	913.28 (731.41, 1131.84)	841.67 (674.54, 1044.47)	-0.48 (-0.63, -0.34)	4810.79 (3870.01, 6010.23)	4525.72 (3664.04, 5634.06)	-0.43 (-0.59, -0.28)
Age												
<5 years	2.76 (1.75, 3.89)	1.60 (1.16, 1.95)	-1.39 (-1.53, -1.25)	257.98 (167.32, 361.48)	153.68 (116.63, 185.10)	-1.34 (-1.47, -1.21)	260.54 (174.62, 364.26)	226.36 (149.87, 321.42)	-0.47 (-0.49, -0.44)	692.31 (464.24, 968.84)	613.74 (409.97, 863.05)	-0.42 (-0.45, -0.39)
5–9 years	0.33 (0.25, 0.41)	0.22 (0.17, 0.25)	-1.05 (-1.15, -0.94)	51.10 (38.34, 67.33)	39.11 (29.07, 54.85)	-0.81 (-0.83, -0.78)	296.82 (172.56, 459.64)	267.25 (153.98, 414.17)	-0.52 (-0.60, -0.44)	1281.08 (803.68, 1928.49)	1149.31 (715.60, 1732.22)	-0.53 (-0.60, -0.46)
10–14 years	0.27 (0.23, 0.32)	0.22 (0.18, 0.26)	-0.42 (-0.50, -0.34)	53.92 (37.34, 78.05)	46.17 (32.37, 67.84)	-0.51 (-0.56, -0.45)	433.76 (235.41, 758.94)	386.92 (209.75, 680.52)	-0.52 (-0.64, -0.41)	1880.63 (1173.05, 3065.04)	1676.49 (1044.74, 2741.04)	-0.54 (-0.67, -0.42)
15–19 years	0.31 (0.25, 0.36)	0.27 (0.22, 0.30)	-0.39 (-0.43, -0.35)	71.97 (46.90, 115.18)	61.57 (40.75, 99.24)	-0.51 (-0.57, -0.45)	638.74 (355.51, 1104.99)	544.73 (302.84, 947.77)	-0.53 (-0.64, -0.43)	3022.69 (1735.93, 4957.79)	2575.62 (1459.62, 4223.70)	-0.54 (-0.65, -0.42)
20–24 years	0.35 (0.29, 0.40)	0.34 (0.28, 0.38)	-0.08 (-0.13, -0.03)	98.57 (61.68, 160.40)	84.59 (53.05, 136.42)	-0.44 (-0.51, -0.37)	860.17 (468.15, 1383.00)	721.55 (389.45, 1169.70)	-0.51 (-0.60, -0.42)	4636.69 (2580.99, 7439.54)	3855.41 (2127.75, 6285.12)	-0.50 (-0.61, -0.38)
25–29 years	0.43 (0.35, 0.49)	0.47 (0.39, 0.51)	0.25 (0.14, 0.36)	125.82 (74.84, 205.38)	114.00 (71.25, 181.27)	-0.33 (-0.40, -0.25)	1014.65 (599.27, 1623.52)	895.35 (524.28, 1427.42)	-0.46 (-0.55, -0.37)	6212.35 (3540.80, 9413.08)	5401.52 (3025.11, 8213.93)	-0.45 (-0.57, -0.33)
30–34 years	0.56 (0.47, 0.62)	0.64 (0.52, 0.70)	0.52 (0.42, 0.63)	150.25 (91.28, 250.03)	146.46 (92.06, 240.22)	-0.20 (-0.31, -0.08)	1120.10 (596.68, 1803.90)	1065.60 (569.14, 1722.72)	-0.36 (-0.50, -0.22)	7406.07 (4446.43, 12627.33)	7013.84 (4215.99, 11975.24)	-0.35 (-0.53, -0.17)
35–39 years	0.70 (0.59, 0.79)	0.85 (0.70, 0.93)	0.67 (0.59, 0.75)	175.77 (103.87, 283.23)	171.28 (107.67, 268.23)	-0.15 (-0.28, -0.03)	1269.32 (706.66, 2073.04)	1180.67 (658.38, 1922.85)	-0.36 (-0.49, -0.23)	8647.42 (4777.73, 13350.41)	8004.14 (4419.33, 12384.32)	-0.36 (-0.54, -0.18)
40–44 years	0.89 (0.77, 0.99)	1.12 (0.95, 1.22)	0.69 (0.60, 0.79)	190.33 (115.44, 304.76)	193.30 (123.12, 300.25)	-0.02 (-0.13, 0.09)	1368.45 (767.92, 2328.47)	1306.88 (735.05, 2224.21)	-0.23 (-0.34, -0.13)	9163.35 (5556.14, 14403.20)	8809.18 (5316.99, 13876.48)	-0.19 (-0.35, -0.02)
45–49 years	1.17 (1.00, 1.29)	1.47 (1.26, 1.60)	0.81 (0.69, 0.93)	207.07 (130.57, 324.40)	218.86 (144.65, 333.64)	0.16 (0.03, 0.29)	1480.06 (815.31, 2317.55)	1462.84 (802.50, 2298.81)	-0.09 (-0.21, 0.03)	9749.47 (5960.77, 15115.36)	9909.54 (6050.37, 15184.96)	0.01 (-0.17, 0.18)
50–54 years	1.61 (1.38, 1.80)	2.01 (1.73, 2.20)	0.84 (0.69, 0.99)	222.19 (146.28, 344.61)	240.97 (164.19, 365.49)	0.27 (0.15, 0.39)	1515.25 (872.97, 2377.44)	1531.37 (876.76, 2415.04)	-0.02 (-0.13, 0.09)	9977.33 (6022.06, 15546.28)	10493.67 (6240.24, 16243.82)	0.11 (-0.05, 0.27)
55–59 years	2.19 (1.93, 2.45)	2.82 (2.50, 3.05)	1.11 (0.98, 1.23)	235.42 (158.88, 351.25)	261.59 (183.52, 383.02)	0.38 (0.27, 0.50)	1588.97 (920.02, 2538.68)	1619.73 (933.49, 2596.55)	-0.04 (-0.17, 0.08)	9943.05 (6152.16, 15911.32)	10499.71 (6539.30, 16914.88)	0.09 (-0.08, 0.25)
60–64 years	3.10 (2.74, 3.44)	4.13 (3.72, 4.51)	1.12 (1.04, 1.20)	257.14 (181.02, 386.79)	288.04 (213.53, 417.80)	0.43 (0.34, 0.52)	1801.00 (1054.64, 3008.00)	1808.29 (1058.01, 3020.43)	-0.01 (-0.15, 0.14)	10198.22 (6236.90, 15871.57)	10442.00 (6459.67, 16153.22)	0.10 (-0.08, 0.28)
65–69 years	4.69 (4.23, 5.28)	5.60 (4.98, 6.07)	0.89 (0.79, 0.99)	288.09 (210.09, 402.89)	315.89 (236.88, 428.97)	0.38 (0.28, 0.48)	1987.38 (1128.19, 3209.10)	2052.14 (1165.23, 3266.58)	0.00 (-0.18, 0.18)	10624.06 (6908.16, 16008.99)	11121.50 (7300.66, 16629.66)	0.08 (-0.13, 0.28)
70–74 years	6.71 (6.07, 7.68)	8.15 (7.36, 8.82)	0.78 (0.69, 0.87)	307.69 (225.78, 411.44)	338.88 (258.24, 444.64)	0.34 (0.24, 0.44)	1957.16 (1162.98, 3122.85)	1973.06 (1172.28, 3162.36)	-0.07 (-0.27, 0.13)	10659.01 (6243.42, 16615.57)	10884.47 (6425.85, 16786.69)	-0.01 (-0.24, 0.22)
75–79 years	10.66 (9.76, 11.73)	12.65 (11.26, 13.63)	0.70 (0.64, 0.76)	331.26 (260.79, 434.78)	370.00 (296.09, 477.94)	0.33 (0.21, 0.46)	1780.79 (1093.90, 2789.71)	1862.09 (1144.58, 2897.80)	-0.07 (-0.31, 0.16)	9771.17 (6384.34, 14739.91)	10307.28 (6707.47, 15382.79)	-0.03 (-0.31, 0.24)
80–84 years	15.98 (14.15, 17.96)	19.35 (16.63, 21.19)	0.96 (0.83, 1.08)	348.93 (284.48, 440.47)	397.11 (328.31, 489.07)	0.54 (0.41, 0.67)	1714.93 (1089.32, 2438.20)	1802.72 (1154.78, 2555.59)	-0.03 (-0.31, 0.25)	9079.66 (6028.41, 12993.10)	9647.34 (6462.42, 13884.37)	0.00 (-0.31, 0.32)
85–89 years	27.35 (23.73, 30.21)	36.15 (29.62, 39.93)	1.45 (1.26, 1.65)	414.55 (343.49, 497.21)	508.41 (420.59, 605.34)	0.97 (0.82, 1.13)	1717.83 (1097.61, 2485.10)	1816.47 (1159.69, 2627.78)	0.02 (-0.24, 0.28)	8742.47 (5858.81, 12374.97)	9454.01 (6339.94, 13425.52)	0.07 (-0.24, 0.37)
90–94 years	44.28 (36.59, 48.97)	69.08 (53.11, 77.53)	2.09 (1.87, 2.30)	536.36 (449.36, 638.92)	760.84 (617.86, 896.34)	1.57 (1.41, 1.74)	1842.84 (1183.88, 2863.16)	1952.99 (1256.41, 3045.61)	0.10 (-0.13, 0.32)	9091.98 (6056.45, 14190.48)	10045.36 (6732.29, 15524.97)	0.19 (-0.10, 0.47)
95+ years	61.46 (47.42, 69.98)	120.48 (86.07, 138.44)	2.70 (2.51, 2.88)	673.92 (547.57, 802.54)	1157.21 (877.09, 1334.52)	2.14 (1.99, 2.29)	2093.23 (1057.50, 3593.36)	2170.41 (1084.35, 3694.55)	0.10 (-0.08, 0.28)	10340.84 (6399.61, 16297.75)	11233.91 (6886.46, 17558.47)	0.20 (-0.04, 0.44)
21 Regions												
Andean Latin America	4.57 (3.18, 5.25)	2.57 (2.07, 3.96)	-1.79 (-2.41, -1.16)	264.47 (185.69, 322.70)	154.38 (118.78, 208.83)	-1.69 (-2.08, -1.29)	793.86 (633.67, 972.21)	786.95 (629.39, 972.72)	-0.15 (-0.23, -0.07)	4714.12 (3813.01, 5829.64)	4684.23 (3829.90, 5792.57)	-0.18 (-0.28, -0.07)
Australasia	2.75 (2.60, 2.88)	3.90 (3.52, 4.14)	1.25 (0.77, 1.72)	140.69 (122.96, 165.10)	160.74 (141.91, 185.69)	0.41 (0.10, 0.72)	478.34 (379.03, 598.22)	490.55 (393.78, 606.70)	0.04 (-0.05, 0.13)	2939.23 (2361.04, 3630.80)	3036.62 (2445.86, 3735.09)	0.04 (-0.06, 0.13)
Caribbean	6.22 (5.34, 7.62)	5.38 (4.40, 6.92)	-0.24 (-0.81, 0.34)	382.76 (297.54, 511.52)	339.09 (259.75, 453.02)	-0.31 (-0.63, 0.01)	1416.88 (1147.62, 1714.44)	1415.98 (1157.22, 1716.68)	-0.21 (-0.35, -0.07)	7420.57 (6003.74, 9037.16)	7581.78 (6233.15, 9135.93)	-0.16 (-0.31, -0.00)
Central Asia	0.59 (0.54, 0.68)	1.19 (1.04, 1.34)	2.17 (1.85, 2.49)	109.75 (80.74, 149.45)	126.11 (97.00, 165.11)	0.29 (0.14, 0.45)	731.63 (579.91, 911.95)	674.67 (536.46, 837.93)	-0.49 (-0.62, -0.35)	4297.57 (3377.44, 5440.08)	4114.40 (3312.59, 5100.79)	-0.34 (-0.46, -0.22)
Central Europe	1.53 (1.44, 1.59)	1.15 (1.05, 1.25)	-1.03 (-1.47, -0.59)	147.33 (120.40, 186.03)	114.95 (89.62, 150.57)	-0.87 (-1.05, -0.70)	724.49 (581.61, 890.75)	714.42 (579.32, 878.76)	-0.15 (-0.23, -0.08)	4175.44 (3363.38, 5161.50)	4131.02 (3366.97, 5100.70)	-0.16 (-0.26, -0.07)
Central Latin America	2.97 (2.87, 3.06)	3.63 (3.27, 3.97)	1.12 (0.90, 1.34)	178.12 (148.22, 220.57)	197.69 (165.87, 239.18)	0.54 (0.43, 0.65)	906.34 (725.76, 1123.01)	986.65 (797.78, 1218.24)	0.17 (0.02, 0.32)	5059.70 (4106.92, 6317.09)	5600.10 (4558.07, 6858.55)	0.17 (-0.01, 0.36)
Central Sub-Saharan Africa	2.38 (1.21, 3.59)	2.51 (1.15, 3.92)	0.14 (0.09, 0.20)	170.86 (105.87, 259.95)	159.37 (98.28, 220.84)	-0.29 (-0.35, -0.24)	754.71 (603.83, 931.24)	764.12 (612.79, 957.48)	-0.11 (-0.25, 0.03)	4320.45 (3421.47, 5351.59)	4349.82 (3502.97, 5410.92)	-0.16 (-0.29, -0.02)
East Asia	1.64 (1.26, 2.05)	1.19 (0.83, 1.46)	-1.18 (-1.46, -0.89)	215.45 (152.18, 299.85)	179.53 (124.67, 260.78)	-0.59 (-0.71, -0.47)	1476.12 (1179.06, 1824.00)	1451.82 (1153.29, 1798.71)	0.02 (-0.08, 0.12)	9215.40 (7338.23, 11627.00)	9273.72 (7251.38, 11797.48)	0.17 (0.01, 0.32)
Eastern Europe	0.71 (0.69, 0.72)	1.08 (1.01, 1.15)	1.04 (0.66, 1.43)	125.28 (94.99, 168.31)	132.02 (102.37, 173.37)	-0.08 (-0.22, 0.06)	975.63 (785.67, 1205.01)	965.39 (777.67, 1188.65)	-0.26 (-0.44, -0.08)	5041.41 (4098.27, 6241.06)	4983.09 (4074.52, 6119.37)	-0.29 (-0.48, -0.10)
Eastern Sub-Saharan Africa	2.03 (1.42, 2.65)	2.07 (1.46, 2.72)	0.01 (-0.02, 0.04)	149.14 (109.27, 196.78)	138.78 (103.41, 179.01)	-0.35 (-0.43, -0.27)	944.28 (758.15, 1163.72)	854.40 (686.74, 1054.40)	-0.55 (-0.70, -0.39)	4963.26 (4024.35, 6145.61)	4588.15 (3710.10, 5690.21)	-0.49 (-0.66, -0.33)
High-income Asia Pacific	1.50 (1.38, 1.64)	1.13 (0.99, 1.22)	-0.99 (-1.09, -0.89)	85.63 (73.50, 102.61)	65.44 (53.92, 81.20)	-0.95 (-1.00, -0.90)	339.11 (275.00, 419.34)	336.46 (273.14, 415.62)	-0.11 (-0.22, -0.00)	1863.74 (1525.13, 2275.68)	1862.28 (1518.12, 2283.99)	-0.10 (-0.20, 0.01)
High-income North America	3.05 (2.91, 3.14)	7.09 (6.51, 7.42)	2.98 (2.70, 3.26)	183.67 (154.28, 225.38)	274.10 (244.80, 313.91)	1.36 (1.09, 1.63)	797.83 (636.00, 1002.80)	833.64 (673.24, 1040.40)	-0.01 (-0.35, 0.32)	4981.48 (3934.24, 6292.25)	5133.04 (4140.67, 6319.01)	-0.14 (-0.63, 0.35)
North Africa and Middle East	2.25 (1.63, 3.71)	2.62 (2.13, 3.41)	0.95 (0.76, 1.15)	184.24 (134.84, 273.39)	173.65 (137.30, 217.17)	-0.03 (-0.09, 0.04)	890.09 (712.43, 1097.95)	830.81 (670.82, 1029.48)	-0.40 (-0.50, -0.29)	4825.35 (3900.01, 6023.39)	4579.62 (3691.88, 5667.72)	-0.35 (-0.46, -0.23)
Oceania	4.35 (2.81, 6.19)	4.29 (2.83, 5.79)	-0.14 (-0.25, -0.03)	270.14 (186.89, 361.89)	278.04 (195.45, 363.99)	0.01 (-0.08, 0.09)	1296.50 (1029.56, 1616.40)	1329.46 (1063.32, 1644.13)	0.05 (-0.01, 0.11)	8332.76 (6617.90, 10479.22)	8870.22 (7050.43, 11029.50)	0.13 (0.05, 0.22)
South Asia	0.46 (0.33, 0.56)	0.49 (0.36, 0.56)	0.21 (0.14, 0.28)	101.13 (69.68, 142.50)	91.66 (65.29, 128.96)	-0.47 (-0.59, -0.35)	870.30 (692.58, 1084.19)	752.58 (601.50, 934.30)	-0.69 (-0.82, -0.56)	4443.34 (3557.83, 5583.65)	3937.93 (3144.71, 4943.96)	-0.63 (-0.78, -0.48)
Southeast Asia	1.45 (1.09, 1.84)	1.59 (1.22, 2.00)	0.24 (0.19, 0.29)	184.78 (127.63, 262.74)	173.38 (123.68, 245.97)	-0.34 (-0.43, -0.25)	1467.37 (1183.04, 1812.37)	1326.39 (1066.04, 1638.47)	-0.45 (-0.54, -0.36)	8525.47 (6837.70, 10647.29)	8020.12 (6380.60, 9991.11)	-0.35 (-0.46, -0.24)
Southern Latin America	5.72 (5.35, 5.99)	2.53 (2.35, 2.67)	-2.17 (-3.08, -1.26)	236.44 (215.44, 264.14)	135.49 (114.96, 162.79)	-1.57 (-2.10, -1.04)	545.85 (441.14, 671.47)	528.34 (427.08, 652.07)	-0.18 (-0.24, -0.13)	3440.03 (2828.87, 4197.00)	3419.60 (2813.36, 4155.19)	-0.12 (-0.19, -0.06)
Southern Sub-Saharan Africa	5.11 (4.25, 7.16)	9.65 (6.15, 11.39)	2.49 (2.30, 2.67)	274.58 (223.84, 355.66)	423.83 (297.70, 508.11)	1.70 (1.52, 1.89)	948.39 (766.54, 1174.56)	1022.24 (829.24, 1258.73)	0.16 (0.01, 0.31)	5123.33 (4189.48, 6190.93)	5576.08 (4551.82, 6678.57)	0.14 (-0.04, 0.32)
Tropical Latin America	2.11 (1.97, 2.23)	4.52 (4.19, 4.75)	2.81 (2.35, 3.28)	148.73 (121.09, 188.00)	217.38 (187.94, 260.05)	1.36 (1.05, 1.68)	876.42 (704.29, 1070.98)	957.26 (770.67, 1184.67)	0.13 (-0.06, 0.31)	4594.44 (3694.19, 5673.37)	5059.31 (4136.61, 6132.05)	0.10 (-0.12, 0.33)
Western Europe	2.89 (2.76, 2.97)	2.91 (2.67, 3.06)	0.64 (0.45, 0.84)	167.10 (143.66, 200.23)	157.00 (133.97, 190.02)	0.13 (0.00, 0.25)	521.16 (425.71, 644.53)	531.12 (432.25, 654.56)	-0.04 (-0.15, 0.08)	4007.00 (3249.35, 4925.84)	4069.17 (3343.02, 4946.66)	-0.06 (-0.19, 0.06)
Western Sub-Saharan Africa	2.12 (1.26, 2.74)	2.10 (1.53, 2.63)	-0.18 (-0.26, -0.09)	143.26 (98.71, 189.01)	136.14 (100.91, 179.52)	-0.33 (-0.43, -0.24)	848.57 (680.13, 1051.17)	814.97 (657.48, 1008.13)	-0.31 (-0.42, -0.20)	4499.19 (3622.66, 5639.09)	4384.27 (3532.53, 5475.47)	-0.28 (-0.41, -0.15)

EAPC, estimated annual percentage change; SDI, Sociodemographic Index; UI, uncertainty interval. EAPC is expressed as 95% con dence interval, ASMR: Age-Standardized Deaths Rate, ASDR: Age-Standardized Disability-adjusted life years Rate, ASIR: Age-Standardized Incidence Rate, ASPR Age-Standardized Prevalence Rate.

**Figure 1:**
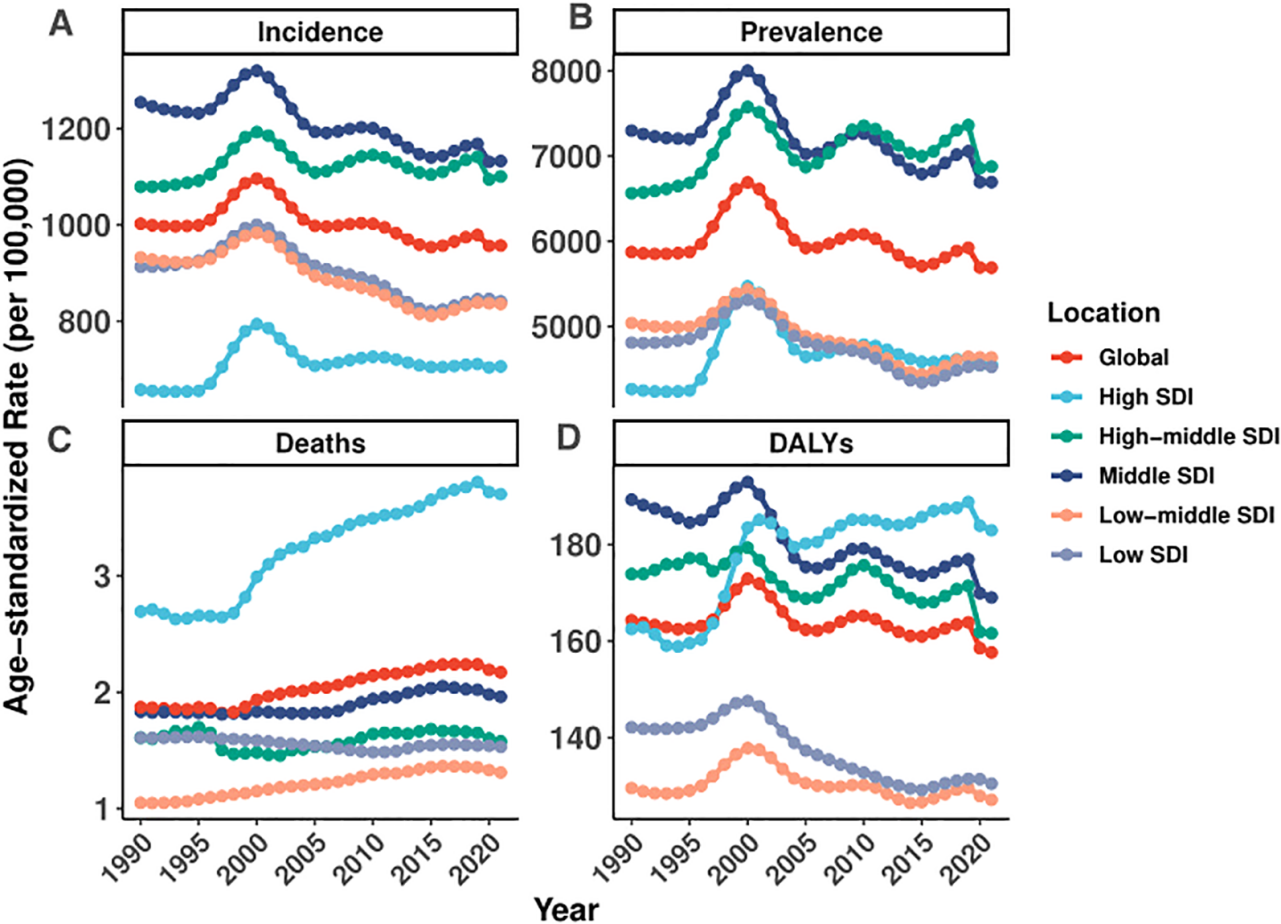
Global and regional trends in age-standardized rates of incidence, prevalence, deaths, and DALYs of EMBID (1990–2021).

Marked disparities were observed across SDI quintiles and regions. High-SDI regions experienced a modest increase in prevalence (EAPC: 0.12% [95% CI: -0.14 to 0.38%]), whereas low-SDI regions exhibited significant declines in both prevalence (EAPC: -0.43% [95% CI: -0.59 – -0.28%]) and incidence (EAPC: -0.48% [95% CI: -0.63 – -0.34%])(**Figure 1**, **Table 1**). Regionally, Southern Latin America (EAPC: -2.17% [95% CI: -3.08 – -1.26%]) and East Asia (EAPC: -1.18% [95% CI: -1.46 – -0.89%]) experienced the most pronounced reductions in ASDR, while Tropical Latin America saw the steepest increase (EAPC: 2.81% [95% CI: 2.35–3.28%]).

### Current distribution in 2021

**Figure 2:**
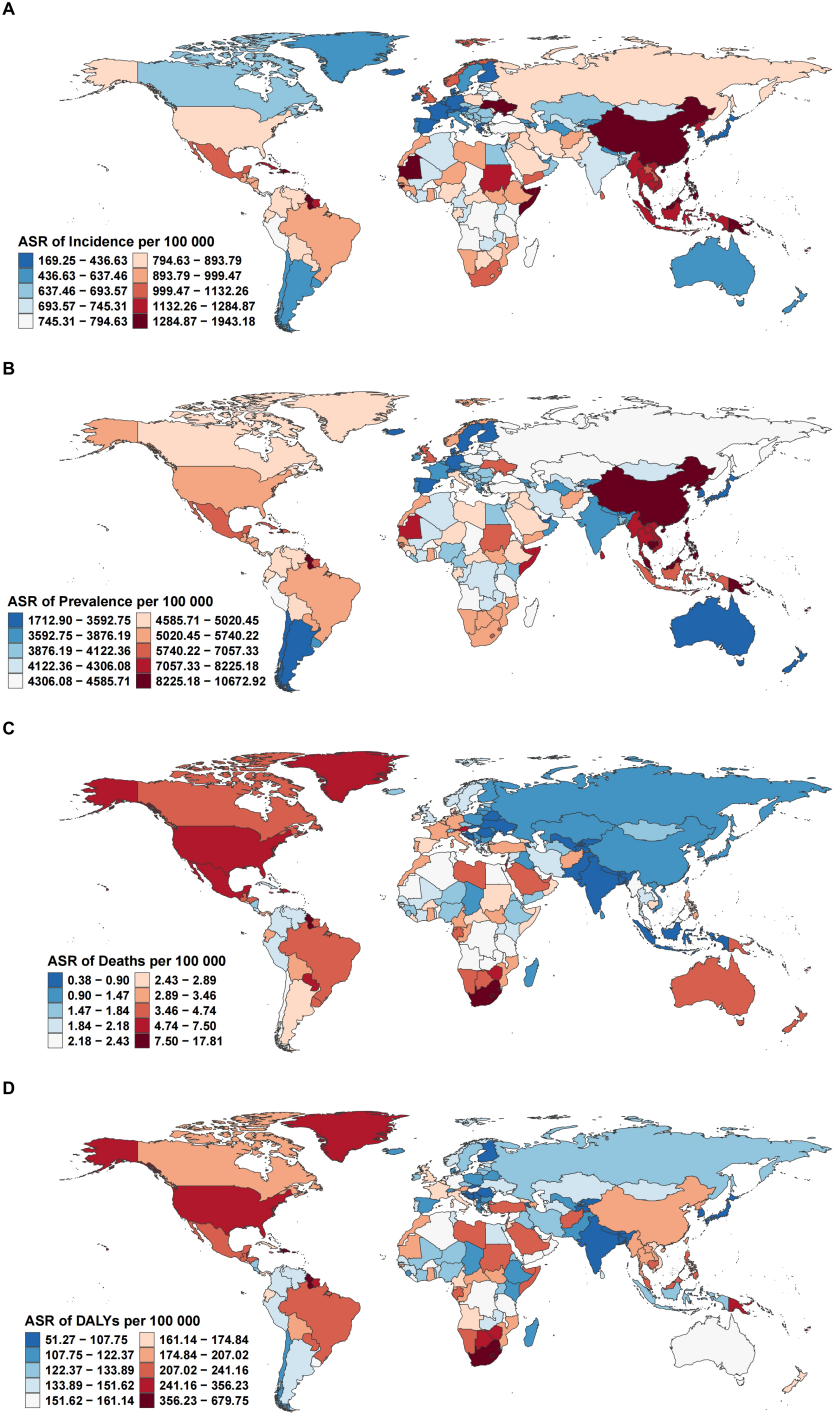
Global distribution of age-standardized rates per 100,000 population for **(A)** incidence, **(B)** prevalence, **(C)** deaths, and **(D)** DALYs of EMBID in 2021.

In 2021, substantial geographical disparities were observed in the ASRs of incidence, prevalence, mortality, and DALYs attributable to EMBID across 204 countries and territories. The highest incidence rates were recorded in the Philippines (1,923.94 per 100,000 [95% UI: 1,556.96–2,353.36]), followed by Haiti and Vanuatu, whereas the lowest were observed in Spain (170.96 [95% UI: 142.04–202.39]), Singapore, and Denmark (**Figure 2A**). In terms of prevalence, Vanuatu (10,567.25 [95% UI: 8,516.56–13,268.97]) and Kiribati exhibited the highest rates, contrasting with the lowest values in Japan, Singapore, and the Republic of Korea (**Figure 2B**).

The greatest mortality burden was observed in American Samoa, which reported the highest age-standardized death rate (17.63 per 100,000 [95% UI: 13.83–23.41]), followed by the Bahamas and South Africa (**Figure 2C**). Conversely, the lowest mortality rates were reported in Andorra, Tajikistan, and Bangladesh. A similar pattern was noted for DALYs, with American Samoa recording the highest DALY rate (673.01 per 100,000 [95% UI: 545.34–849.11]), alongside the Bahamas and Guyana. In contrast, the lowest DALY rates were documented in the Republic of Korea, Singapore, and Andorra (**Figure 2D**).

### Temporal trends by region and SDI level

**Figure 3:**
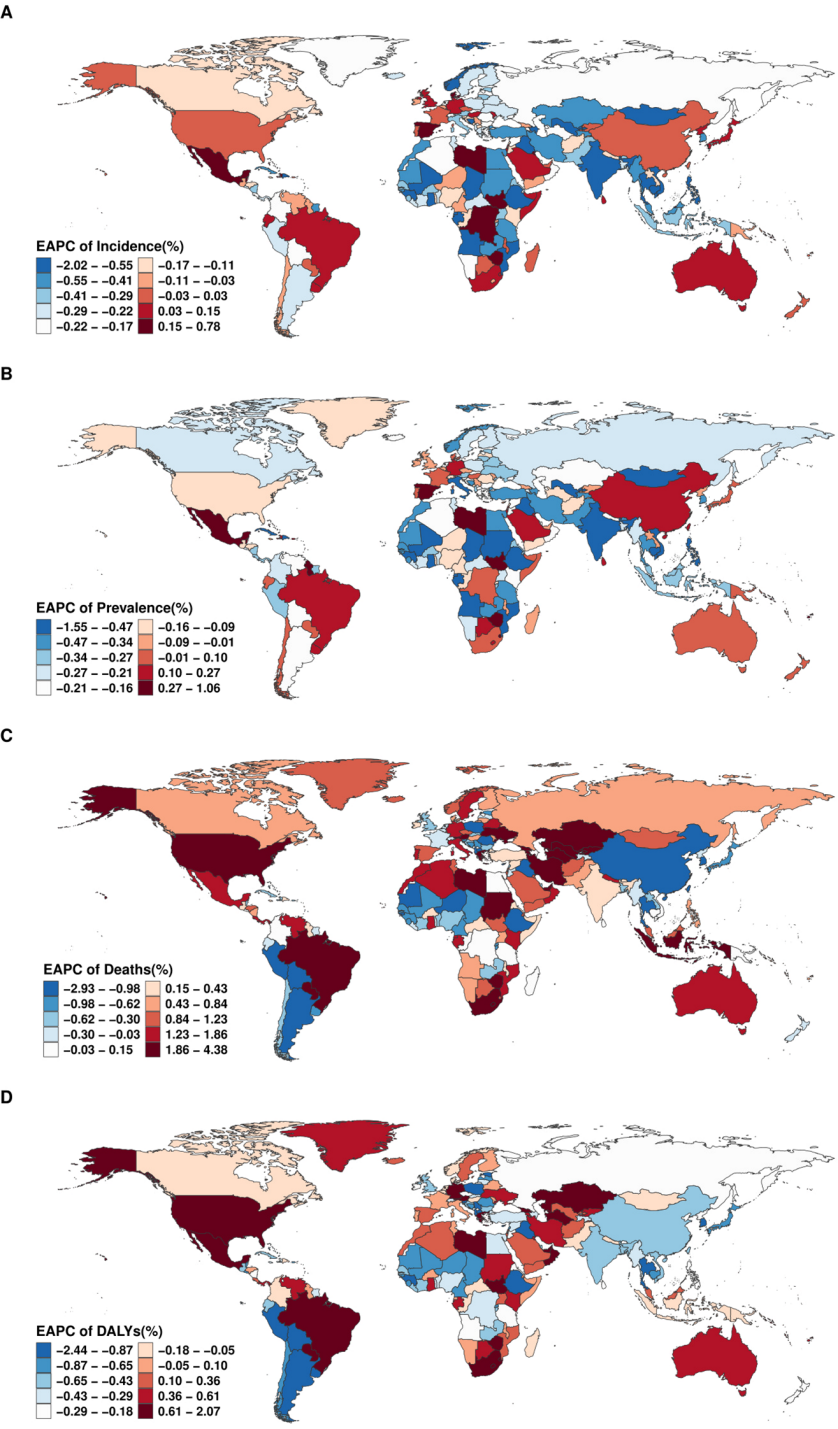
Global distribution of the EAPC for age-standardized rates of **(A)** incidence, **(B)** prevalence, **(C)** deaths, and **(D)** DALYs of EMBID from 1990 to 2021.

From 1990 to 2021, country-level trends in the burden of EMBID demonstrated marked heterogeneity across incidence, prevalence, mortality, and DALYs. The largest increases in ASIR were recorded in Spain (EAPC: 0.77% [95% CI: 0.56–0.98%]), while the steepest declines occurred in Equatorial Guinea (-2.00% [95% CI: -2.30 – -1.70%])(**Figure 3A**). For ASPR, American Samoa (1.05% [95% CI: 0.92–1.17%]) exhibited the fastest growth, whereas Equatorial Guinea (-1.53% [95% CI: -1.76 – -1.31%]) experienced the largest decreases (**Figure 3B**). In terms of ASMR, the most pronounced increases in ASMR were observed in Georgia (EAPC: 4.33% [95% CI: 3.49–5.18%]), while Peru (-2.90% [95% CI: -3.61 – -2.19%]) experienced the greatest reduction (**Figure 3C**). Trends in DALYs mirrored these patterns, with the highest increases reported in Lesotho (2.05% [95% CI: 1.81–2.28%]), while Peru (-2.41% [95% CI: -2.86 – -1.96%]) and Cyprus (-1.84% [95% CI: -1.94 – -1.74%]) showed the largest declines (**Figure 3D**).

### Association with socio-demographic index

**Figure 4:**
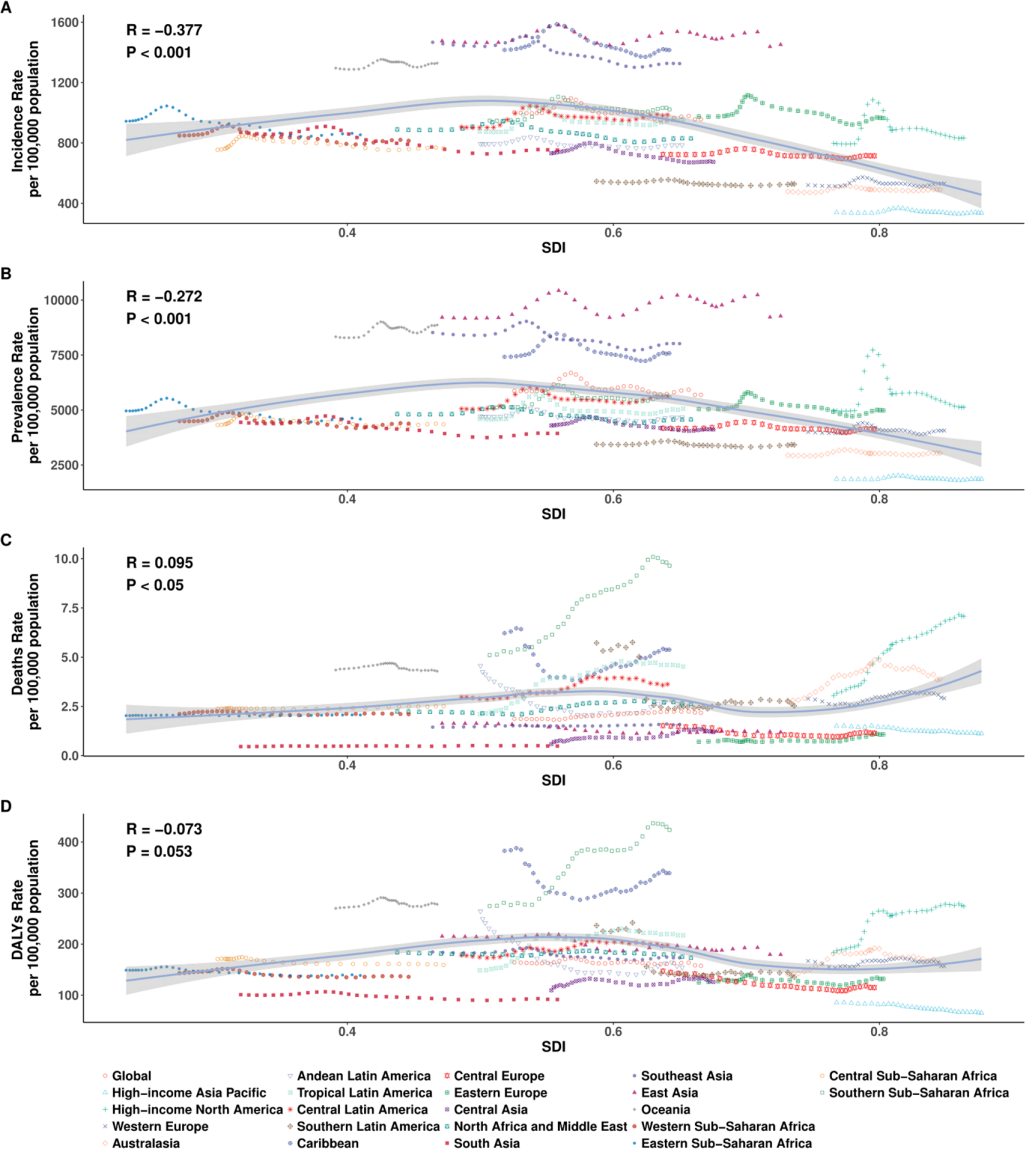
Age-specific number and age-standardized rates per 100,000 population of **(A)** incidence, **(B)** prevalence, **(C)** deaths, and **(D)** DALYs of EMBID by sex in 2021. Data are shown with 95% uncertainty intervals (UIs).

In 2021, the relationship between the burden of EMBID and socio-demographic development, as measured by the SDI, demonstrated diverse patterns across the 21 GBD regions. A moderate negative correlation was observed between SDI and the ASIR (R = -0.377, p < 0.001), with lower-SDI regions such as Oceania and Western Sub-Saharan Africa exhibiting the highest ASIRs, exceeding 1,600 per 100,000 population (**Figure 4A**). In contrast, high-SDI regions, including High-income Asia Pacific and Western Europe, displayed markedly lower ASIRs, typically below 400 per 100,000. A similar but weaker inverse association was observed between SDI and the ASPR (R = -0.272, p < 0.001), with the highest ASPRs concentrated in Southeast Asia, South Asia, and Oceania (**Figure 4B**).

At the regional level (21 SDI regions), the ASMR demonstrated a weak positive correlation with SDI (R = 0.095, p < 0.05), indicating that high-SDI regions such as Central Europe and High-income Asia Pacific may experience higher mortality burdens. Conversely, regions with lower SDI, including South Asia and Eastern Sub-Saharan Africa, recorded lower ASDRs, potentially reflecting diagnostic limitations and underreporting. The association between SDI and the ASDR was negative but not statistically significant (R = -0.073, p = 0.053), although DALY rates remained notably high in low-SDI regions such as Central Sub-Saharan Africa and Oceania (>600 per 100,000), while East Asia and Eastern Europe reported rates below 200 per 100,000 (**Figures 4C, D**).

**Figure 5:**
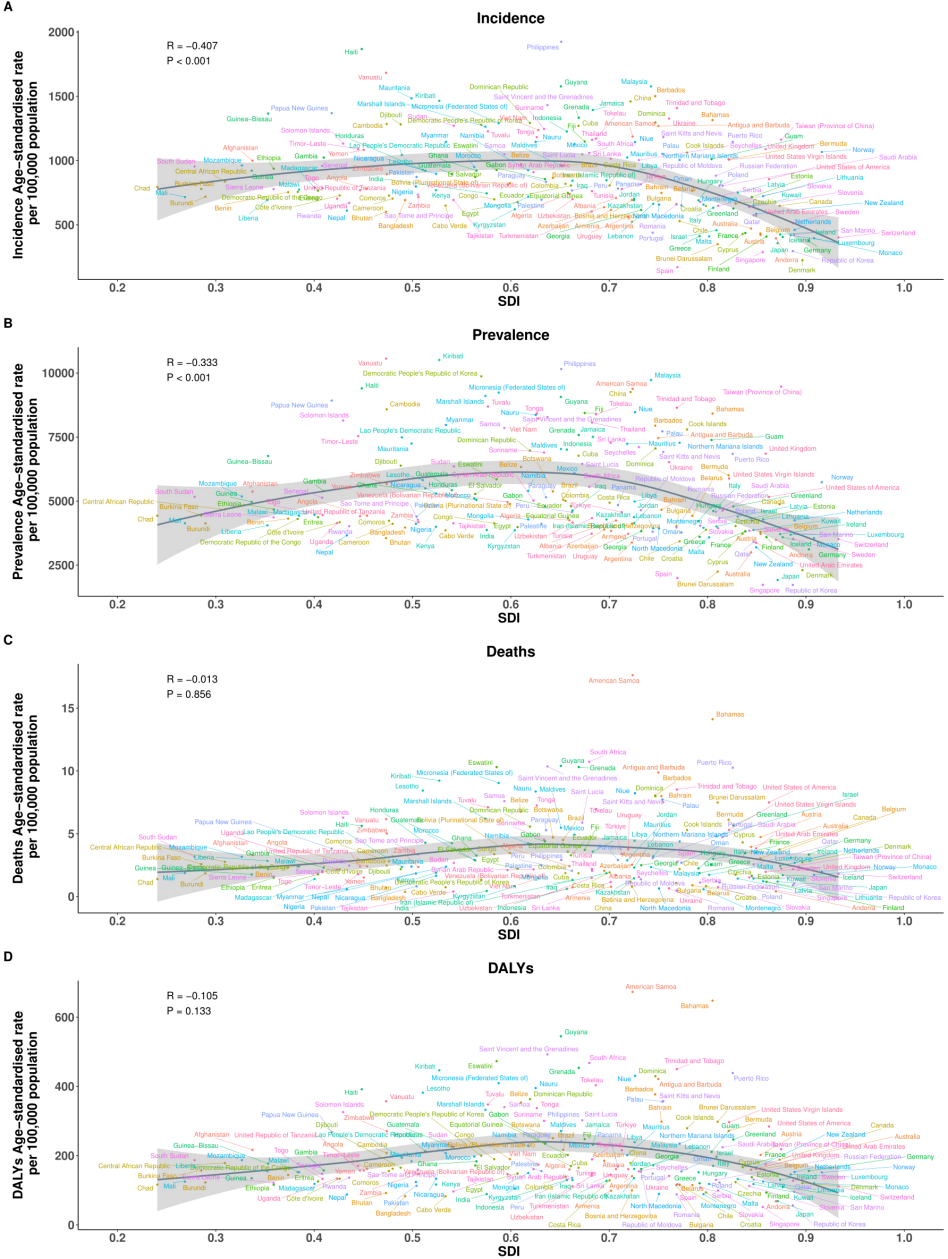
Association between the SDI and age-standardized rates per 100,000 population of **(A)** incidence, **(B)** prevalence, **(C)** deaths, and **(D)** DALYs across the 21 Global Burden of Disease regions.

However, at the national level (204 countries), analysis of 204 countries reinforced these patterns, with significant negative correlations between SDI and both ASIR (R = -0.407, p < 0.001) and ASPR (R = -0.333, p < 0.001), indicating that countries with lower socio-demographic development tend to bear a higher frequency of EMBID. In contrast, no significant associations were observed between SDI and either ASMR (R = -0.013, p = 0.856) or ASDR (R = -0.105, p = 0.133) (**Figures 5A-D**). Notably, several low-SDI countries, including Ethiopia, Nigeria, and the Democratic Republic of the Congo, displayed disproportionately high ASIRs and ASPRs, while high-SDI countries such as Japan, the Republic of Korea, and Western Europe consistently exhibited the lowest burden estimates, particularly for incidence and prevalence.

### Age- and sex-specific patterns

**Figure 6:**
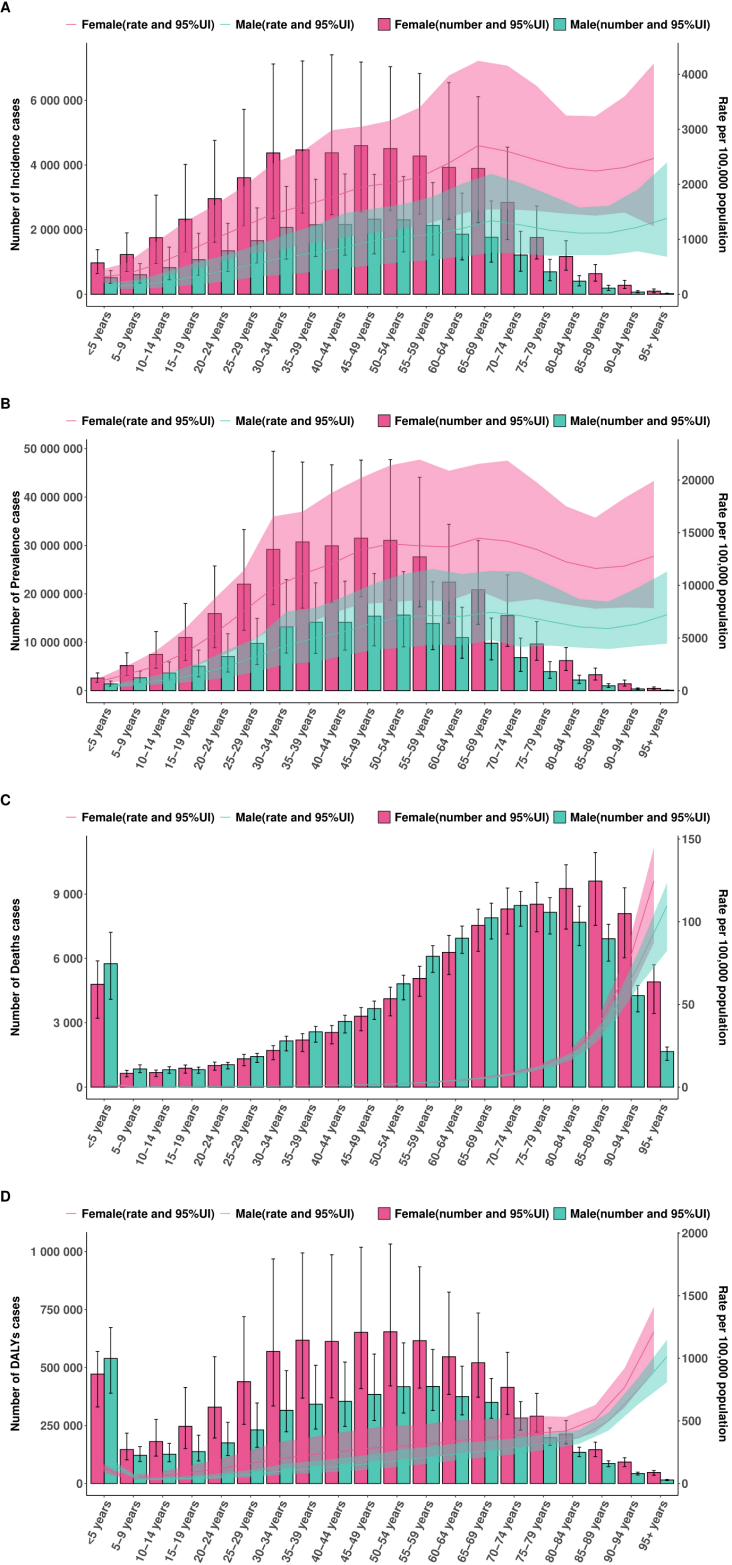
Association between the SDI and age-standardized rates per 100,000 population of **(A)** incidence, **(B)** prevalence, **(C)** deaths, and **(D)** DALYs across 204 countries and territories in 2021.

In 2021, the burden of EMBID exhibited distinct age- and sex-specific patterns across all four key metrics. Incidence and prevalence rates increased progressively with age, with females consistently demonstrating higher rates than males across most age groups. Among females, the highest ASIR was observed in the 65–69 age group, reaching 2,703.62 per 100,000 population (95% UI: 1,537.95–4,246.47). In contrast, the peak ASIR among males occurred in those aged ≥95 years, at 1,382.72 per 100,000 (95% UI: 682.47–2,401.14). Similarly, ASPR were highest among females aged 65–69 years, reaching 14,481.16 per 100,000 (95% UI: 9,520.07–21,537.67), while the corresponding peak for males was 7,451.55 per 100,000 (95% UI: 4,823.09–11,385.16) (**Figures 6A, B**). Conversely, ASMR and ASDR were consistently higher in males, particularly among older age groups. Among individuals aged ≥95 years, males exhibited an ASMR of 109.72 per 100,000 (95% UI: 82.41–123.65) and ASDR of 1,014.58 (95% UI: 809.35–1,150.67) (**Figures 6C, D**). In comparison, females of the same age group demonstrated an ASDR of 124.61 per 100,000 (95% UI: 87.29–144.81) and an age-standardized DALY rate of 1,211.97 (95% UI: 902.98–1,413.96).

### Age–Period–Cohort effects

**Figure 7:**
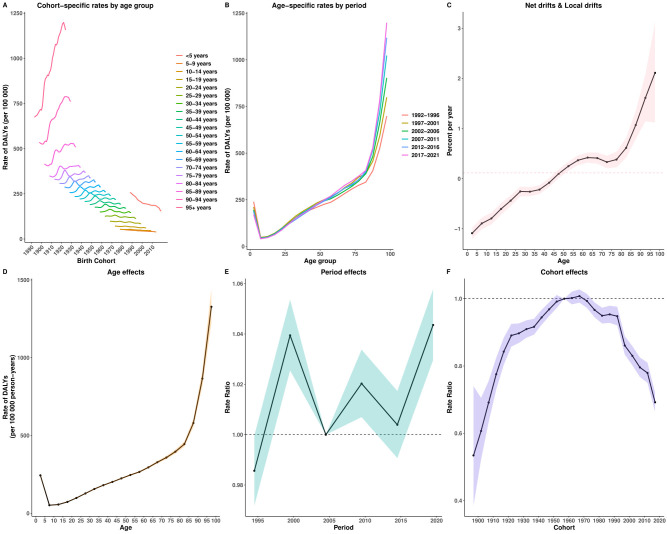
Age–Period–Cohort analysis of age-standardized rates for DALYs of EMBID. Panels show **(A)** cohort-specific rates by age group, **(B)** age-specific rates by period, **(C)** net drift and local drifts, **(D)** age effects, **(E)** period effects, and **(F)** cohort effects.

The APC analysis revealed distinct age, period, and cohort effects in the burden of EMBID. ASIR increased with age, peaking among individuals aged ≥95 years (2,159.25 per 100,000; 95% CI: 1,929.45–2,416.43) (**Supplementary Figure S2A**). ASPR was highest among those aged 70–75 years (11,385 per 100,000; 95% CI: 11,116–11,660) (**Supplementary Figure S3A**). The net drift analysis indicated a slight global decline in incidence (-0.23% per year; 95% CI: -0.28% – -0.18%) and prevalence (-0.172% per year; 95% CI: -0.228% – -0.117%) (**Supplementary Figure S1C**). ASDR demonstrated an exponential increase with age, reaching 174.43 per 100,000 among individuals aged ≥95 years (95% CI: 168.11–180.99) (**Supplementary Figure S1A**). Global mortality showed a modest overall increase (0.68% per year; 95% CI: 0.62%–0.73%). Local drift analysis revealed declining mortality among younger populations (<40 years), but increasing rates among those aged ≥40 years, peaking at 2.78% (95% CI: 2.42%–3.14%) among those aged ≥95 years. DALYs also showed an exponential increase with age, peaking at approximately 1,000 per 100,000 in individuals aged ≥95 years (**Figure 7A**). The net drift analysis indicated a gradual global increase in DALYs (0.115% per year; 95% CI: 0.063%–0.167%). Local drift analysis highlighted a decrease in DALY rates among younger populations (<50 years) but a rise among older adults, particularly among those aged 90–95 years (1.61% per year; 95% CI: 1.14%–2.08%).

Period effects displayed fluctuating risks, peaking around 1999 and stabilizing in recent years. Cohort effects indicated a higher disease burden among earlier birth cohorts (1950s), followed by a gradual decline in subsequent cohorts, particularly among those born after 2000. Among these, the 2017 birth cohort showed significantly reduced risks (relative risk: DALYs = 0.692, 95% CI: 0.664–0.722; incidence = 0.893, 95% CI: 0.872–0.915; prevalence = 0.912, 95% CI: 0.894–0.930).

### Decomposition analysis

**Figure 8:**
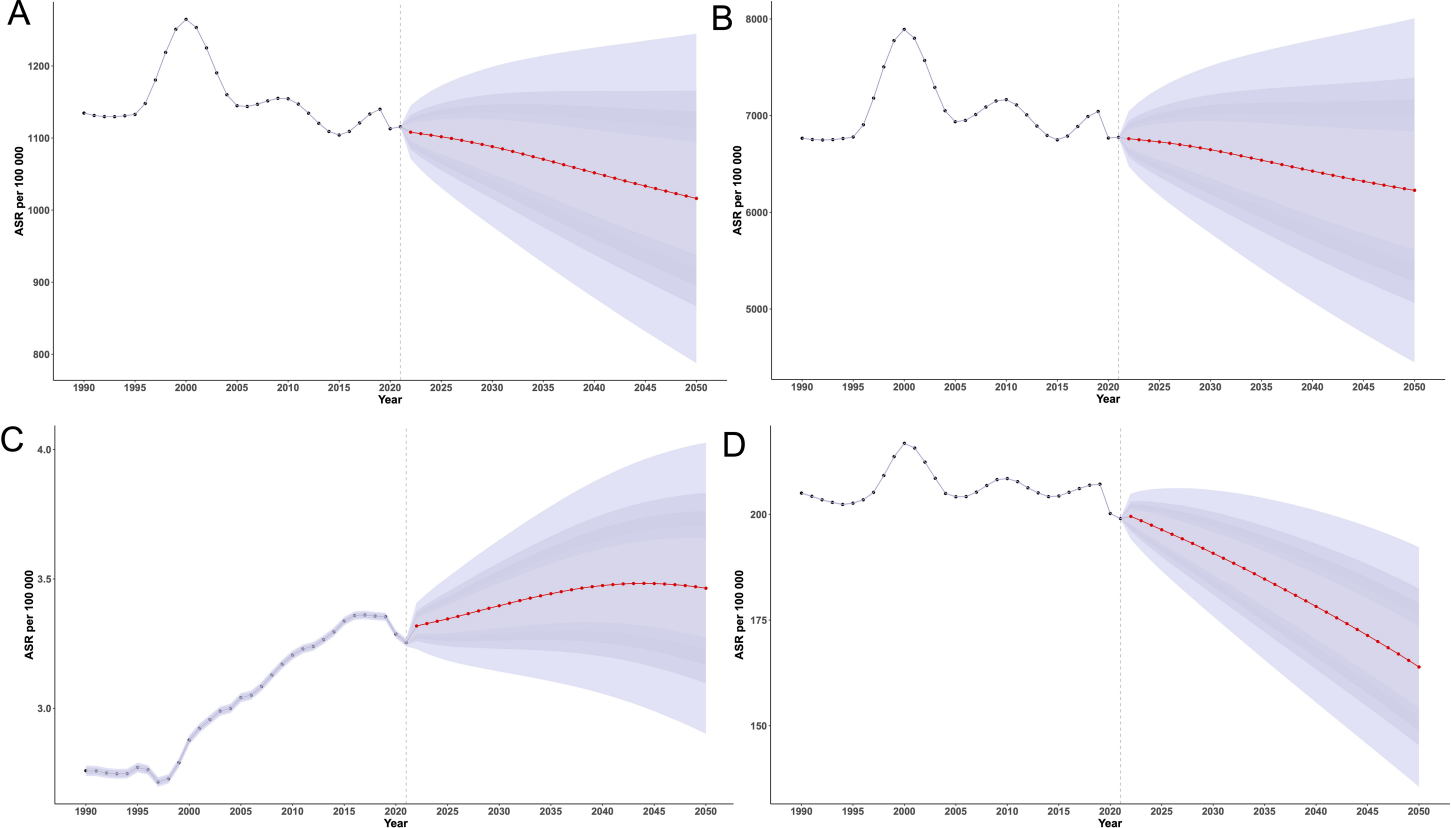
Decomposition analysis of the change in **(A)** incidence, **(B)** prevalence, **(C)** deaths, and **(D)** DALYs of EMBID from 1990 to 2021, showing contributions from population growth, population aging, and epidemiological changes, stratified by sex and SDI levels.

Between 1990 and 2021, the global DALYs due to EMBID increased by 4.68 million, primarily driven by population growth (85.83%) and aging (26.02%), partially offset by a reduction due to epidemiological improvements (-11.86%) (**Figure 8D**). Deaths rose by 97,457 cases, largely attributed to population growth (47.60%) and aging (37.51%), with a smaller positive contribution from adverse epidemiological factors (14.89%) (**Figure 8B**). Incidence increased by 30.38 million cases, mainly due to population growth (81.82%) and aging (29.60%), despite a decrease from epidemiological improvements (-11.42%) (**Figure 8A**). Prevalence grew by 190.83 million cases, driven by population growth (78.40%) and aging (35.44%), partially mitigated by a decline in epidemiological factors (-13.84%) (**Figure 8A**).

Regionally, high and high-middle SDI regions demonstrated substantial epidemiological improvements, moderating demographic pressures, particularly in DALYs and deaths. In contrast, low-middle and low SDI regions experienced substantial increases in all metrics, predominantly driven by aging and population growth, with minimal or negative contributions from epidemiological change. Middle SDI regions exhibited the most pronounced negative epidemiological impacts, particularly in DALYs and prevalence. Female populations consistently showed greater absolute increases in disease burden across all regions.

### Future projections to 2050

**Figure 9:**
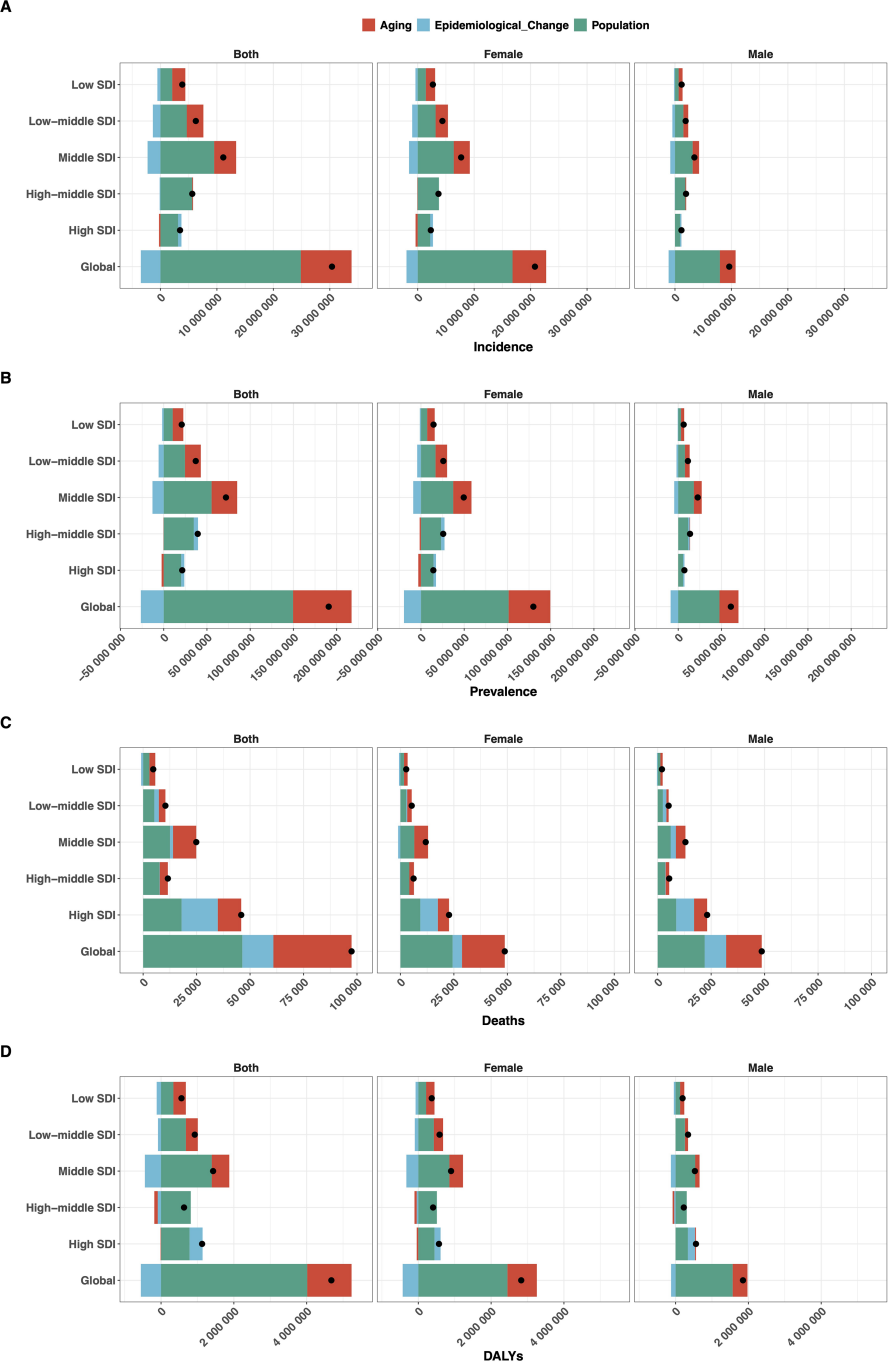
BAPC model projections of age-standardized rates per 100,000 population for **(A)** incidence, **(B)** prevalence, **(C)** deaths, and **(D)** DALYs of EMBID from 1990 to 2050.

In 2021, the global ASIR of EMBID was 1,115.65 per 100,000 population (95% UI: 1,115.38,1,115.93), and is projected to decline to 1,016.21 (95% UI: 787.60,1,244.83) by 2050 (**Figure 9A**). The ASPR showed minor fluctuations and is expected to decrease from 6,775.41 per 100,000 in 1990 (95% UI: 6,774.73,6,776.08) to 6,227.61 (95% UI: 4,445.50,8009.71]) in 2050. The global ASDR increased from 2.76 per 100,000 in 1990 (95% UI: 2.74,2.78) to a peak of 3.36 in 2017 (95% UI: 3.34,3.38), then declined slightly to 3.25 in 2021 (95% UI: 3.24,3.27), and is projected to rise again to 3.47 (95% UI: 3.06,3.89) by 2040. The age-standardized DALY rate is projected to decline from 199.01 per 100,000 in 2021 (95% UI: 198.89,199.12) to 163.9 (95% UI: 135.58,192.23) in 2050.

## Discussion

The present study provides a comprehensive assessment of the global burden and temporal trends of EMBID from 1990 to 2021, highlighting significant disparities by age, sex, geography, and socio-demographic development. Despite overall improvements in healthcare systems globally, demographic shifts—particularly aging populations and population growth—have resulted in substantial increases in disease burden across key metrics, particularly in middle and low-middle SDI regions. These findings are consistent with previous research showing that regions with limited healthcare access, inadequate preventive services, and insufficient public health infrastructure experience disproportionately higher burdens of chronic diseases (18, 19). Strengthening healthcare systems, enhancing health education, and expanding preventive services are essential for addressing these disparities (20, 21).

While this study assessed EMBID as an aggregated disease group, it is important to highlight the substantial contribution of major metabolic and endocrine conditions—particularly diabetes mellitus, obesity, and thyroid dysfunction—to the overall burden. These conditions are among the most prevalent and clinically impactful within the EMBID category, and their trajectories largely drive the trends observed in incidence, mortality, and DALYs. For example, diabetes alone accounts for a significant proportion of EMBID-related deaths and disability, especially in low- and middle-income settings. Acknowledging the central role of diabetes, obesity, and thyroid disorders within the EMBID category enhances the interpretability and clinical significance of the reported trends.

The global ASDR and age-standardized DALY rate have exhibited divergent trends. While mortality modestly increased, likely reflecting aging populations and improved survival from other causes of death (22, 23), DALY rates slightly declined, suggesting enhancements in disease management and treatment efficacy (24, 25). This paradox aligns with previous studies indicating that improved survival and prolonged disease duration may increase chronic disease prevalence despite stable or slightly reduced incidence (26, 27). For instance, advancements in diagnostic technologies, the availability of effective therapies, and the integration of chronic disease management programs have improved survival outcomes even as the overall prevalence of chronic conditions has risen. These findings emphasize the importance of maintaining a balance between extending life expectancy and ensuring quality of life through comprehensive chronic disease management.

The observed higher burden of disease among males, especially in mortality and DALYs, aligns with prior research identifying gender-specific differences in disease susceptibility, healthcare-seeking behavior, and access to healthcare services. Men are more likely to engage in high-risk behaviors such as smoking, excessive alcohol consumption, and poor dietary habits, which contribute to elevated mortality rates (28, 29). In contrast, females consistently exhibited higher incidence and prevalence rates, particularly among middle-aged and older individuals, which may reflect longer life expectancy, greater susceptibility to autoimmune and metabolic disorders, and gender disparities in healthcare utilization (30). These differences underscore the importance of developing gender-sensitive health policies, improving access to diagnostic and treatment services, and promoting health education tailored to specific population groups.

As presented in the results, females showed higher incidence and prevalence of EMBID, whereas males experienced higher mortality across most SDI regions. This sex-specific disparity likely stems from biological, behavioral, and sociocultural factors. Biologically, females are more prone to autoimmune and endocrine diseases—such as thyroiditis and lupus—due to estrogen-mediated immune enhancement and X-linked gene expression (31, 32). Behaviorally, men are more likely to engage in health-risk behaviors and delay seeking care, often resulting in more severe disease and higher mortality, particularly from cardiometabolic conditions (33). Socioculturally, women in low- and middle-income countries may face barriers to healthcare due to limited autonomy and health literacy, while men may underutilize services due to stigma around help-seeking (34, 35). To address these disparities, gender-sensitive strategies—such as equitable screening, tailored health education, and provider training—are essential to improve early detection and reduce sex-based inequalities in outcomes.

Our findings reveal substantial geographical variations in the burden of EMBID, with low- SDI countries disproportionately affected by higher incidence and prevalence rates, consistent with previous studies (36–38). Regions such as Oceania, South Asia, and Sub-Saharan Africa bear the highest burden, reflecting healthcare inequities, limited access to preventive services, and chronic underinvestment in health infrastructure (39, 40). These regions often face a dual burden of infectious and non-communicable diseases, further straining healthcare systems and limiting the effectiveness of chronic disease management.

Conversely, high-SDI regions showed improved disease metrics, reflecting effective preventive strategies, early diagnosis, and comprehensive chronic disease management (41, 42). However, notable disparities persist even within high-SDI regions; Central Europe and high-income Asia-Pacific demonstrated elevated mortality burdens, highlighting the impact of demographic aging and variable healthcare capacities (10, 43). These findings underscore the need for targeted interventions that account for regional healthcare capacities, emphasizing preventive strategies, chronic disease management, and equitable resource allocation.

The decomposition analysis reveals that population aging and growth are the primary drivers of the increasing global burden of EMBID, outweighing the beneficial effects of epidemiological improvements in incidence and prevalence (44, 45). These demographic pressures are most pronounced in middle-SDI regions, where rapid population growth and aging intersect with limited healthcare capacity, creating a significant public health challenge (44). As populations age, the cumulative impact of chronic disease risk factors, coupled with reduced physiological resilience, leads to a substantial increase in disease burden among older adults (45).

The adverse epidemiological impact observed in middle-SDI regions underscores the critical need for strengthening chronic disease management systems and integrating robust prevention strategies into healthcare policies (46, 47). These regions often face a dual burden of persistent infectious diseases and rising non-communicable diseases, further straining healthcare resources. Limited access to quality healthcare, inadequate diagnostic capabilities, and insufficient health education exacerbate the burden of EMBID in these settings (46). To mitigate the impact of demographic pressures, healthcare systems in middle-SDI regions must prioritize chronic disease prevention, enhance primary healthcare infrastructure, and ensure equitable access to diagnostic and treatment services. Policy interventions should focus on promoting healthy aging, scaling up preventive healthcare, and strengthening the healthcare workforce to meet the growing demand for chronic disease management.

Our APC analysis further elucidates the complex interplay of demographic and epidemiological dynamics underlying the burden of EMBID. The observed cohort effects, characterized by increased risks among earlier birth cohorts followed by notable improvements in younger cohorts, reflect the substantial impact of public health interventions, enhanced healthcare access, and lifestyle modifications in recent decades (48, 49). These positive trends among younger cohorts may be attributed to widespread health education, improved maternal and child healthcare, expanded vaccination programs, and the early detection and management of chronic conditions (50).

However, the rising burden among the oldest age groups highlights a contrasting challenge. As life expectancy increases, the accumulation of age-related comorbidities, frailty, and reduced physiological resilience among older adults has contributed to a disproportionate rise in disease burden (51). This demographic shift underscores the urgent need for targeted healthcare strategies tailored to the needs of aging populations, including the integration of geriatric care, multimorbidity management, and comprehensive chronic disease prevention (52, 53). Strengthening primary healthcare, enhancing access to age-appropriate diagnostic and therapeutic services, and training healthcare professionals in geriatric medicine are critical measures to address the growing burden among the elderly.

Projections up to 2050 suggest a complex future landscape, with declining incidence and prevalence rates of EMBID, potentially reflecting the successful implementation of preventive strategies and health education globally (53, 54). These declines may be attributed to enhanced public health initiatives, widespread screening programs, improved diagnostic capabilities, and increased health literacy, which have collectively contributed to early detection and management of chronic conditions (55). For instance, the adoption of lifestyle modification programs targeting obesity, hypertension, and diabetes has proven effective in many high-SDI regions, where robust healthcare infrastructure facilitates widespread implementation (54, 55). While the BAPC model provides a robust framework for long-term projections, its predictions are subject to several important assumptions. These include the continuation of historical trends in incidence and mortality, the stability of age, period, and cohort effects, and the accuracy of population forecasts. The model assumes that no major external shocks—such as emerging pandemics, dramatic shifts in healthcare systems, or large-scale policy changes—will disrupt existing patterns (56, 57). Additionally, demographic assumptions such as population growth and aging trajectories are based on the most recent UN estimates, which may vary over time (15). These uncertainties could lead to either overestimation or underestimation of the projected burden. Although we did not conduct multi-scenario modeling in this study, future research should consider incorporating best-case, worst-case, or policy-intervention scenarios to provide a more comprehensive view of potential outcomes (58).

Nevertheless, the projected increase in mortality rates among aging populations signifies persistent challenges related to chronic disease management, resource allocation, and healthcare system adaptation to demographic changes (59, 60). The aging population is particularly vulnerable to multimorbidity, frailty, and age-related declines in immune function, making them more susceptible to severe outcomes from EMBID. In low- and middle-SDI regions, healthcare systems may struggle to provide age-appropriate care, with gaps in geriatric services, limited access to advanced treatments, and insufficient healthcare workforce exacerbating these challenges (61, 62). Even in high-SDI regions, the growing number of older adults places increasing pressure on healthcare systems, necessitating a shift towards integrated care models that emphasize chronic disease management, palliative care, and coordinated geriatric services (62, 63). High-quality chronic disease management systems, characterized by enhanced diagnostic capacities, personalized treatment protocols, and comprehensive care coordination, remain crucial for mitigating future disease burden. Sustained public health campaigns promoting healthy lifestyles, early diagnosis, and adherence to medical treatments can further reduce disease incidence and improve outcomes.

This study offers a comprehensive assessment of the global burden and temporal trends of EMBID from 1990 to 2021, utilizing standardized methodologies from the GBD Study 2021. The application of consistent analytical frameworks across 204 countries and territories, along with age- and sex-specific estimates, ensures comparability over time and across regions. Advanced statistical models, such as the CODEm for mortality and the Bayesian meta-regression tool DisMod-MR 2.1 for non-fatal outcomes, enhance the robustness of the findings by integrating diverse data sources.

## Limitation

This study has several limitations. First, although the GBD framework employs standardized and validated methodologies, the accuracy of disease burden estimates is constrained by the quality and availability of primary data. In low-SDI countries, underreporting, incomplete surveillance, and poor-quality health records are common, potentially leading to biased estimates—particularly for mortality and prevalence—and affecting cross-regional comparability. Misclassification and underdiagnosis in resource-limited settings further exacerbate these challenges. Second, several key determinants of disease burden—such as environmental exposures, behavioral risks, healthcare access, socioeconomic status, and policy implementation—were not directly accounted for. Their exclusion may result in over- or underestimation and limits the capacity to fully explain regional disparities. Third, this analysis treated EMBID as an aggregated Level 2 cause group, consistent with the GBD framework. While this enables global comparability, it masks disease-specific trends and heterogeneity in etiology and severity. Future research should prioritize condition-specific modeling to improve interpretive precision and inform targeted interventions.

## Conclusion

Reducing the EMBID burden in low-SDI regions requires multisectoral approaches focused on health system strengthening, equitable care access, and sustained public health action. Priority should be given to low-cost, scalable interventions such as community-based screening for diabetes, anemia, and thyroid disorders using point-of-care tools; mobile or radio-based health education; and training of community health workers for basic NCD care and follow-up. Integrating these services into existing primary care systems—rather than building parallel infrastructures—has proven effective in resource-limited settings. Notably, WHO’s Package of Essential Noncommunicable Disease Interventions (PEN) and Rwanda’s CHW-led screening programme offer real-world models for implementation.

## Data Availability

The original contributions presented in the study are included in the article/**Supplementary Material**. Further inquiries can be directed to the corresponding author.

## Supplementary material

Supplementary Figure 1 Age–Period–Cohort analysis of age-standardized rates for Deaths of EMBID. Panels show **(A)** cohort-specific rates by age group, **(B)** age-specific rates by period, **(C)** net drift and local drifts, **(D)** age effects, **(E)** period effects, and **(F)** cohort effects.

Supplementary Figure 2 Age–Period–Cohort analysis of age-standardized rates for Incidence of EMBID. Panels show **(A)** cohort-specific rates by age group, **(B)** age-specific rates by period, **(C)** net drift and local drifts, **(D)** age effects, **(E)** period effects, and **(F)** cohort effects.

Supplementary Figure 3 Age–Period–Cohort analysis of age-standardized rates for Prevalence of EMBID. Panels show **(A)** cohort-specific rates by age group, **(B)** age-specific rates by period, **(C)** net drift and local drifts, **(D)** age effects, **(E)** period effects, and **(F)** cohort effects.
