# Supplementary figures and images for: Global, regional, and national burden of endocrine, metabolic, blood, and immune disorders from 1990 to 2021, and projections to 2050: a systematic analysis of the global burden of disease study
Source: Front Endocrinol (Lausanne). 2025 Jul 25;16:1631123. doi: 10.3389/fendo.2025.1631123 (PMC12331491; doi:10.3389/fendo.2025.1631123)

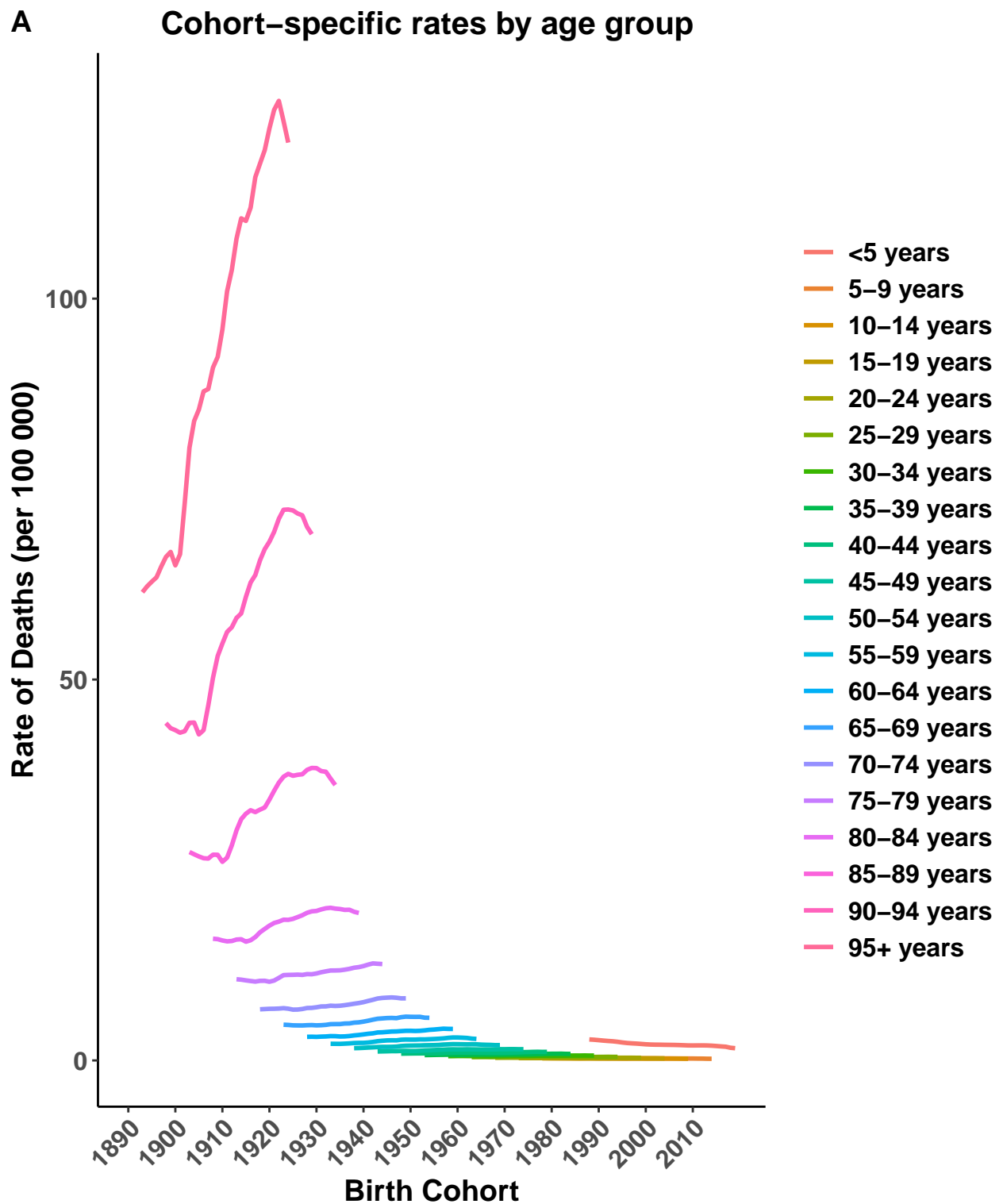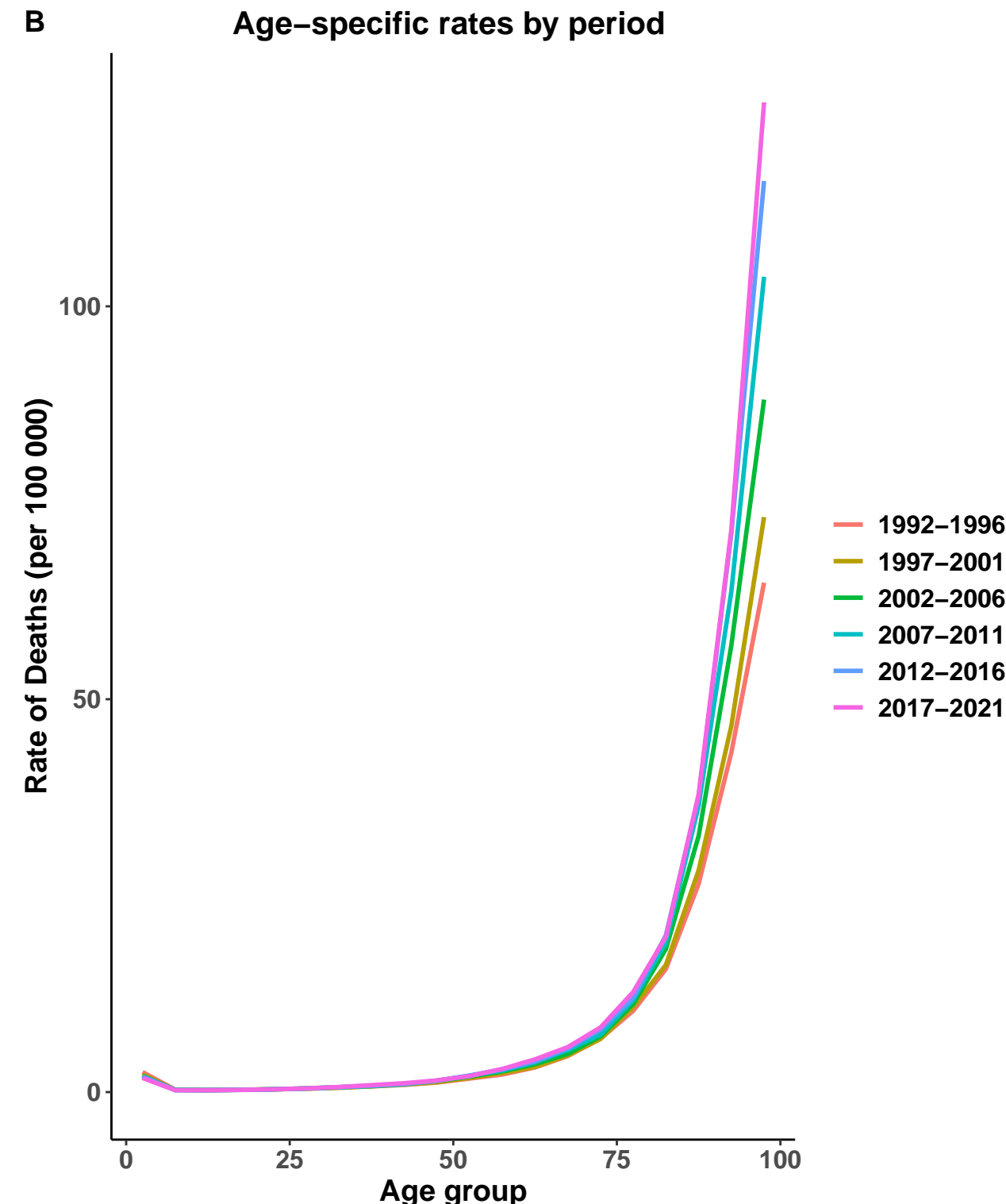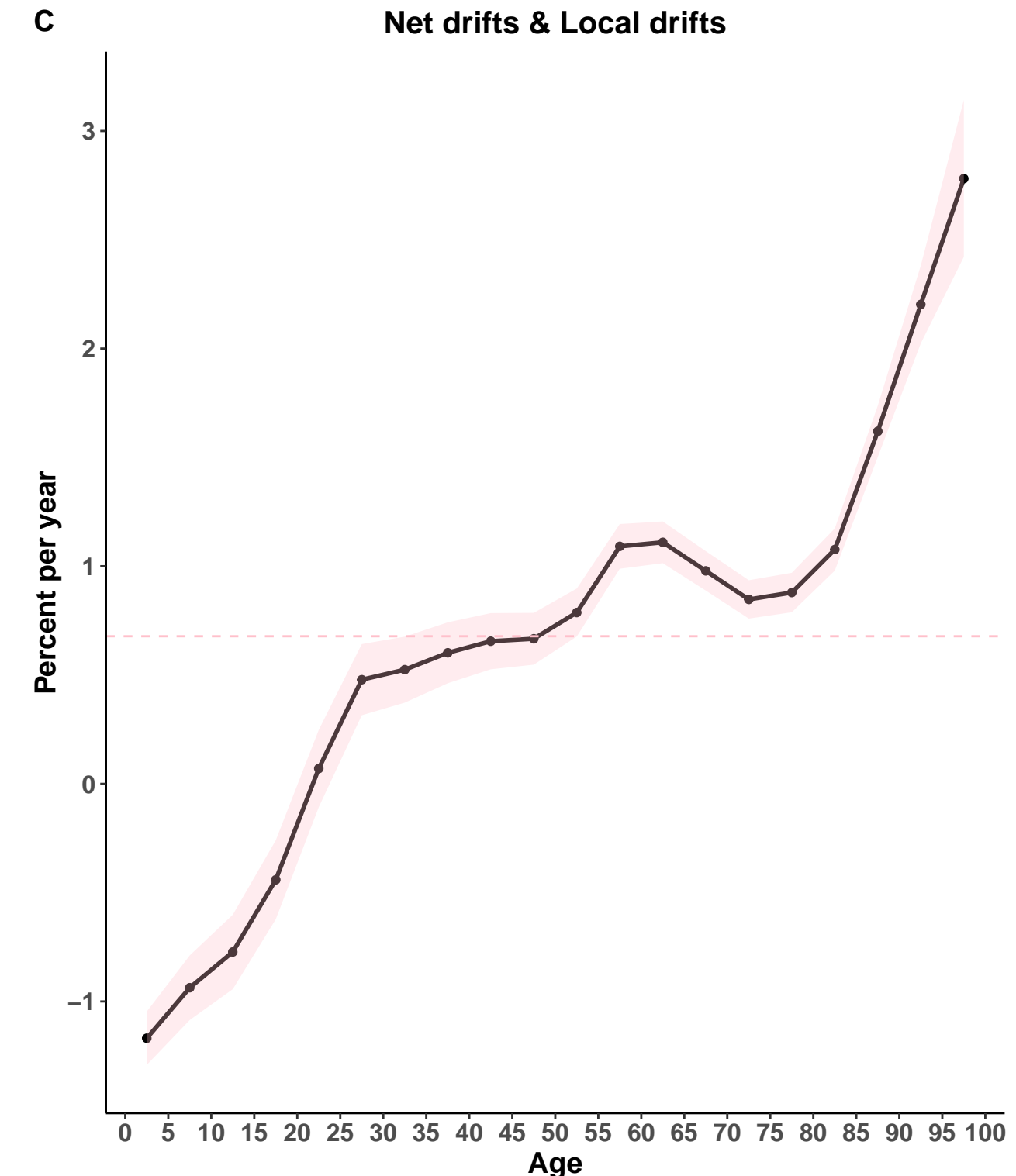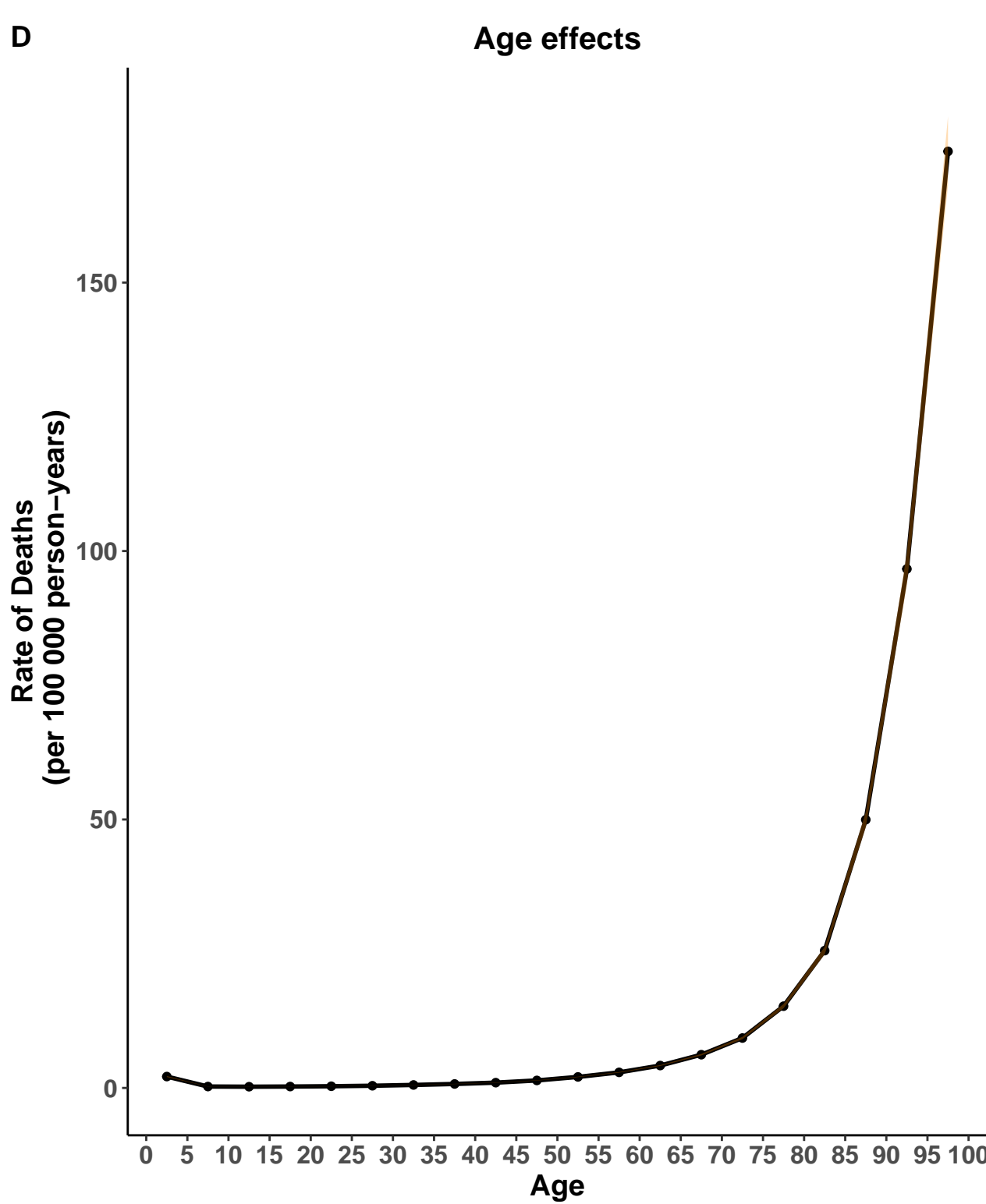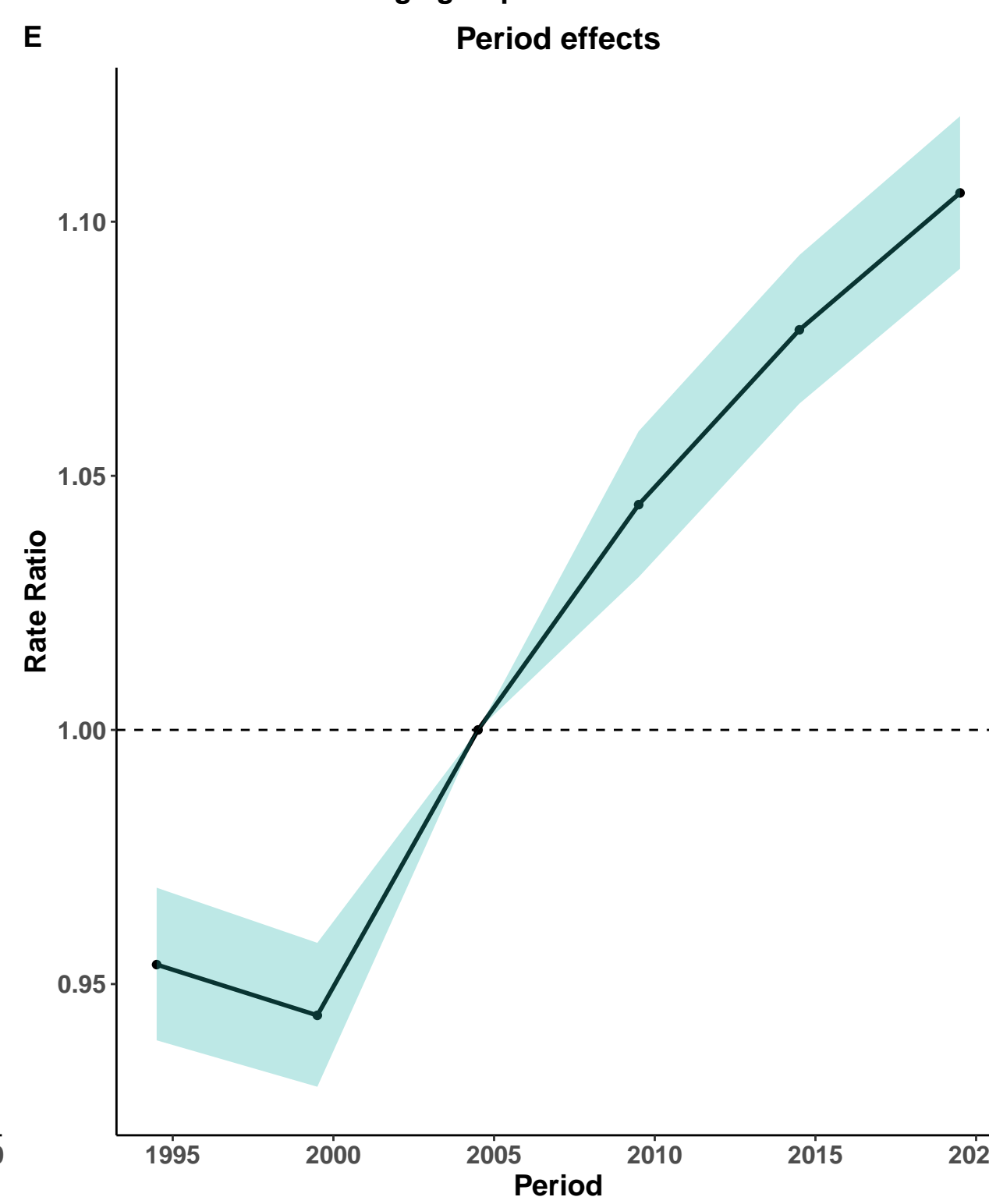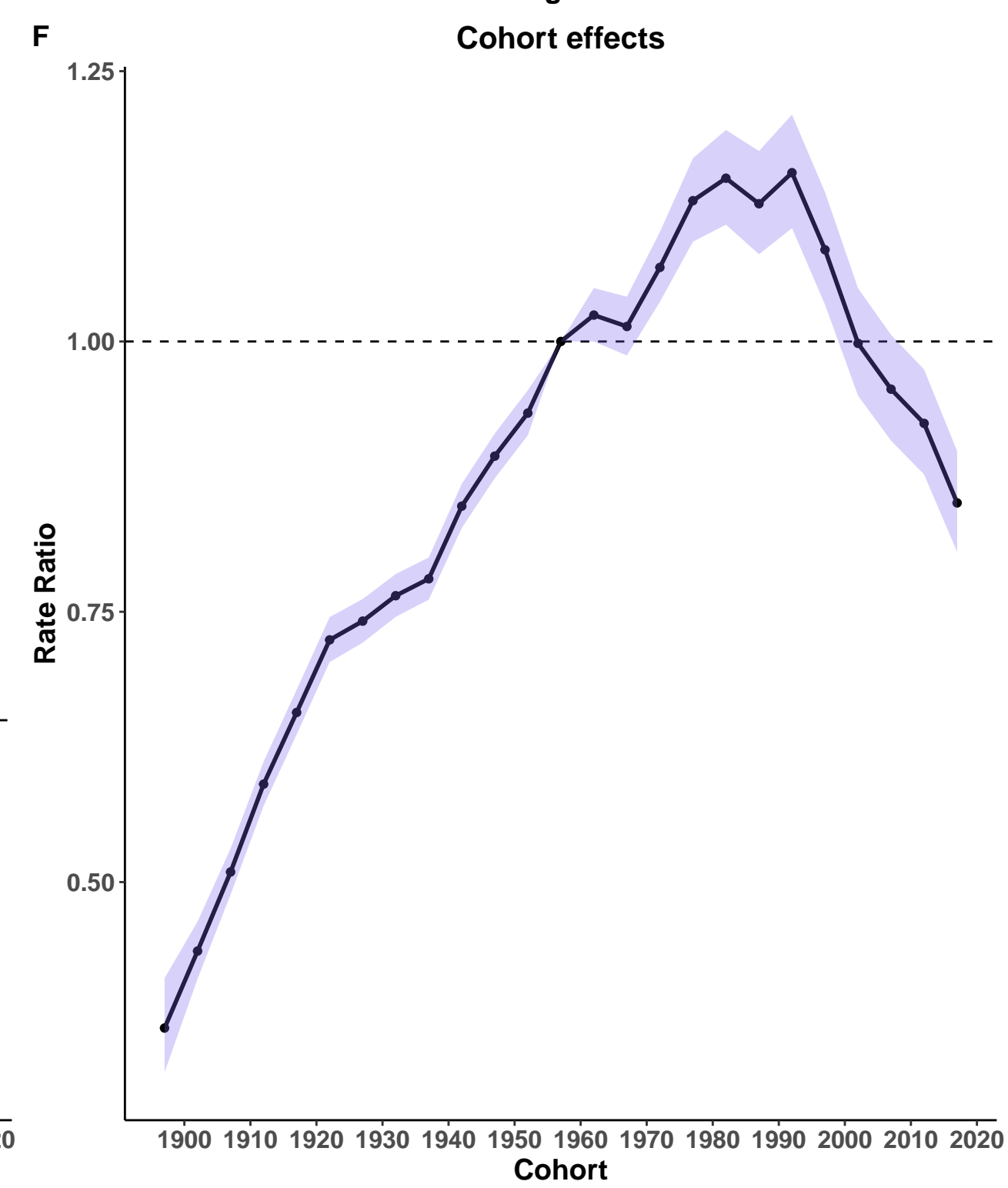

Supplement: Supplementary Figure 1 — Age–Period–Cohort analysis of age-standardized rates for Deaths of EMBID. Panels show (A) cohort-specific rates by age group, (B) age-specific rates by period, (C) net drift and local drifts, (D) age effects, (E) period effects, and (F) cohort effects. [file DataSheet1.pdf]

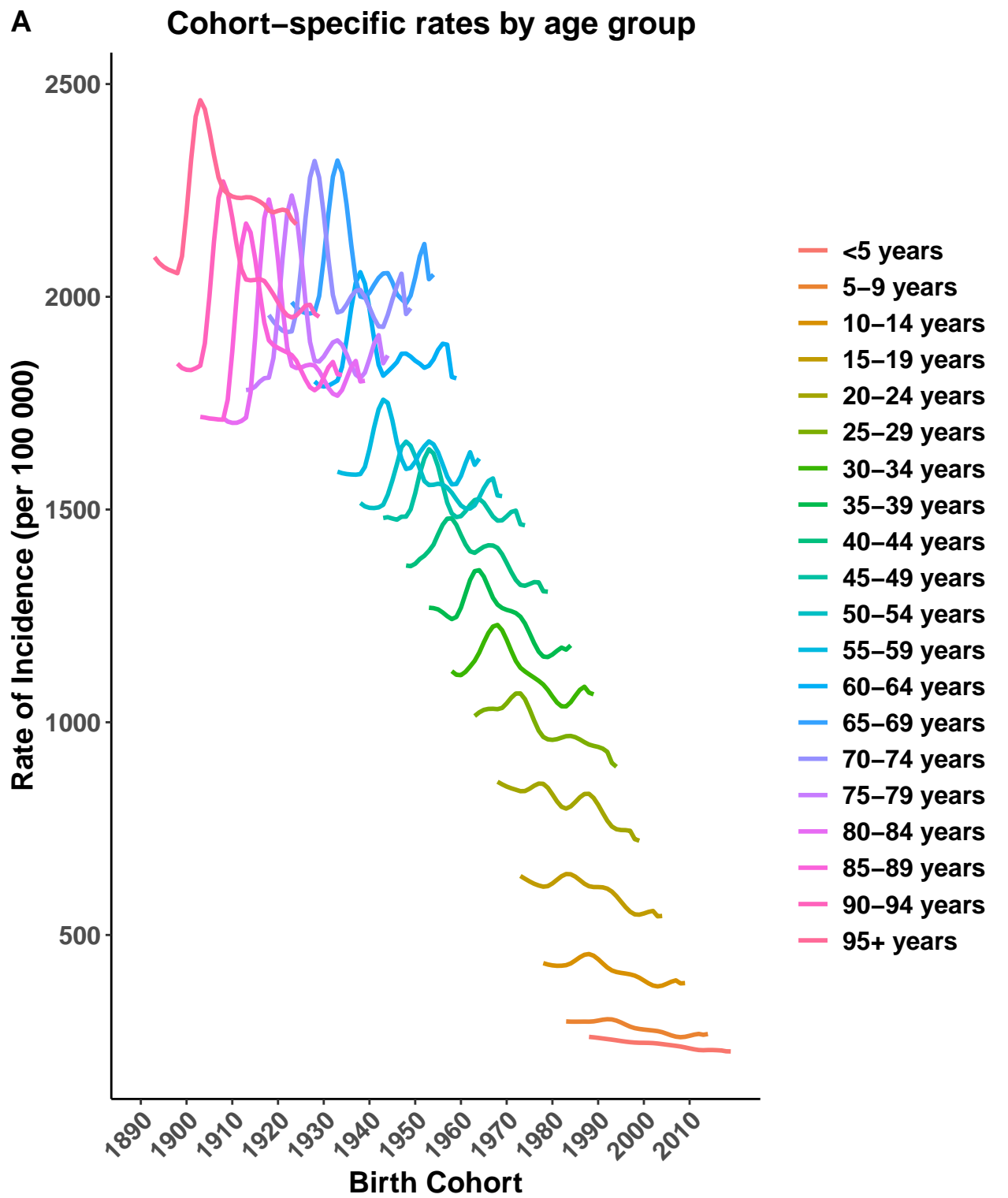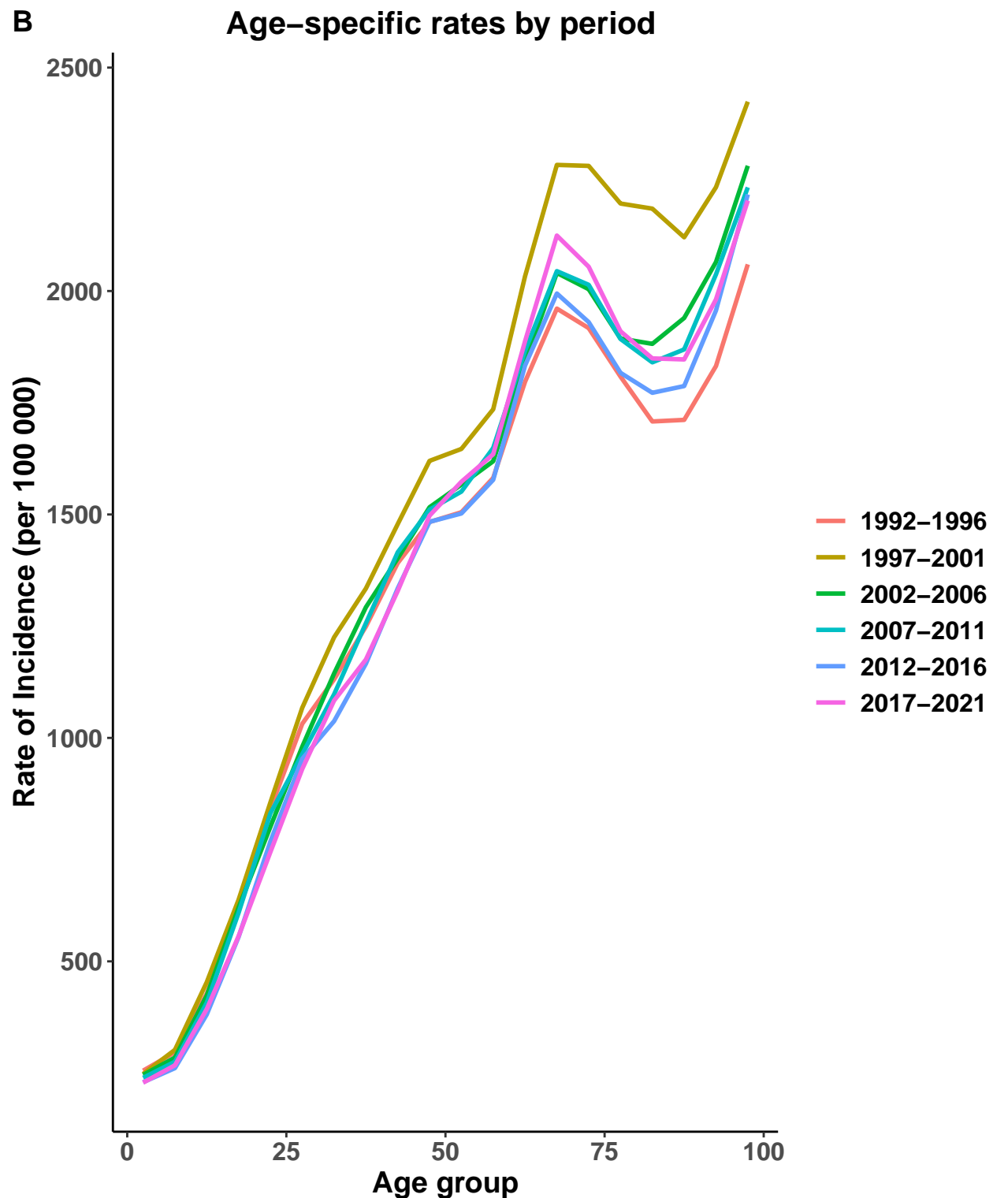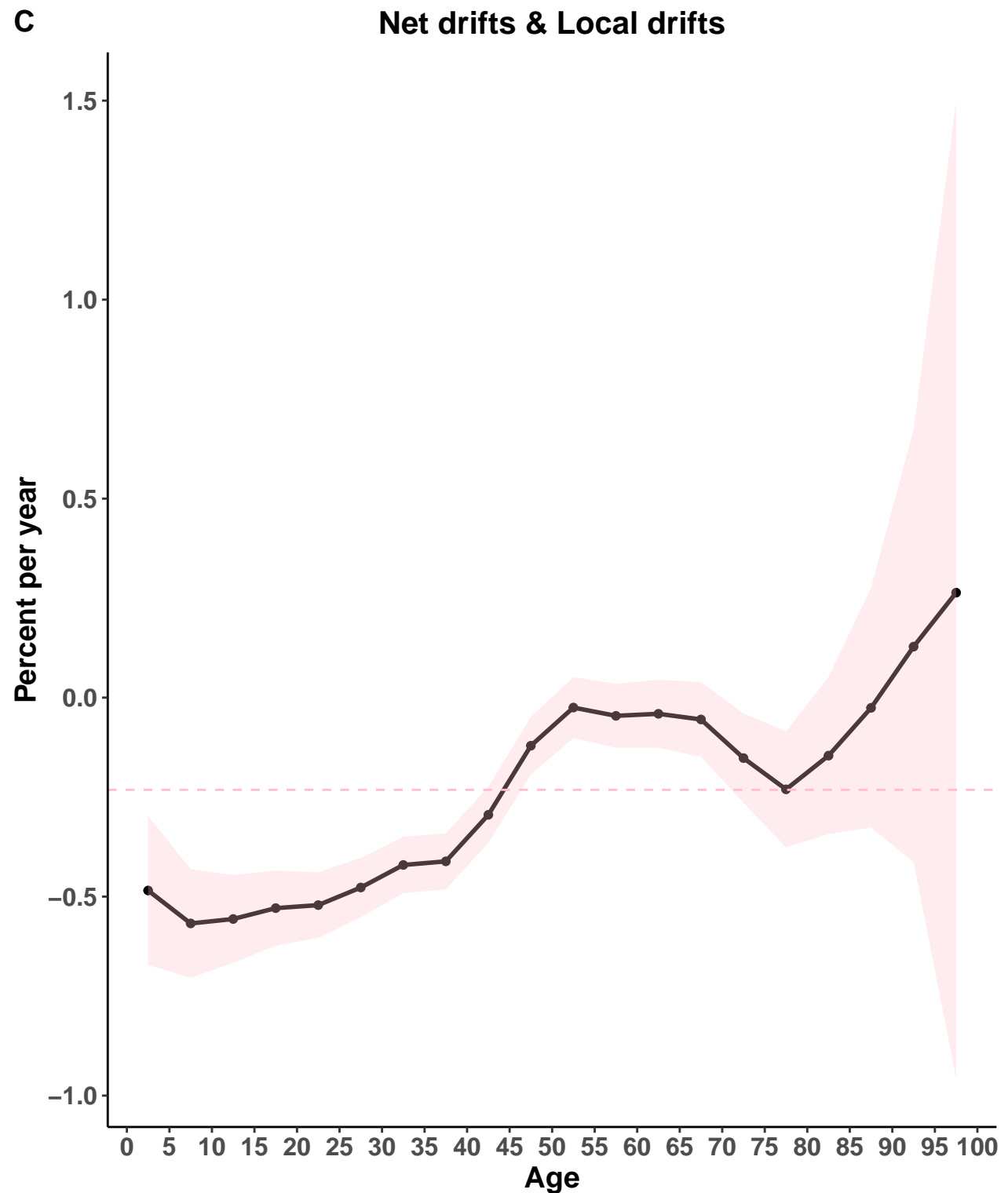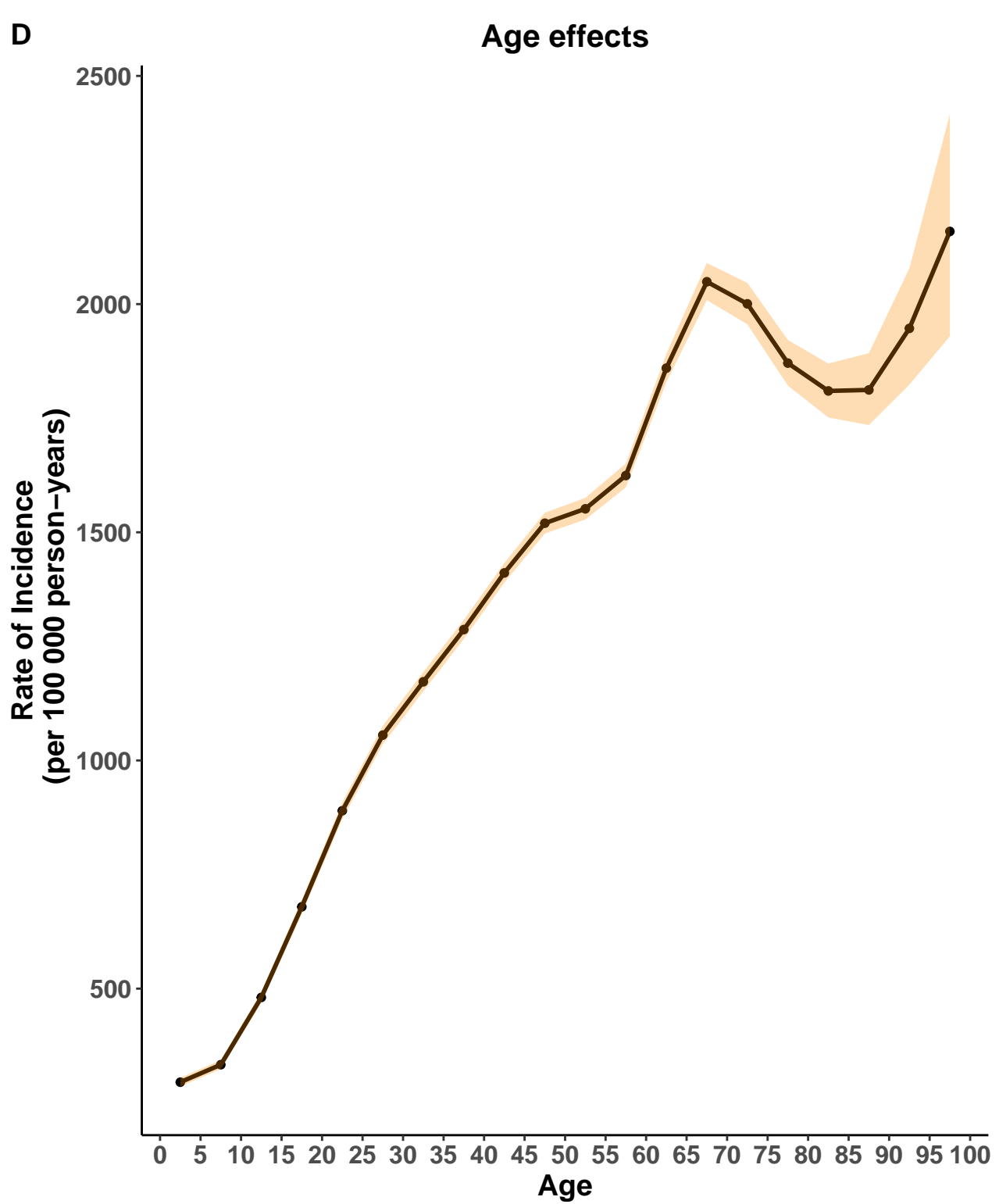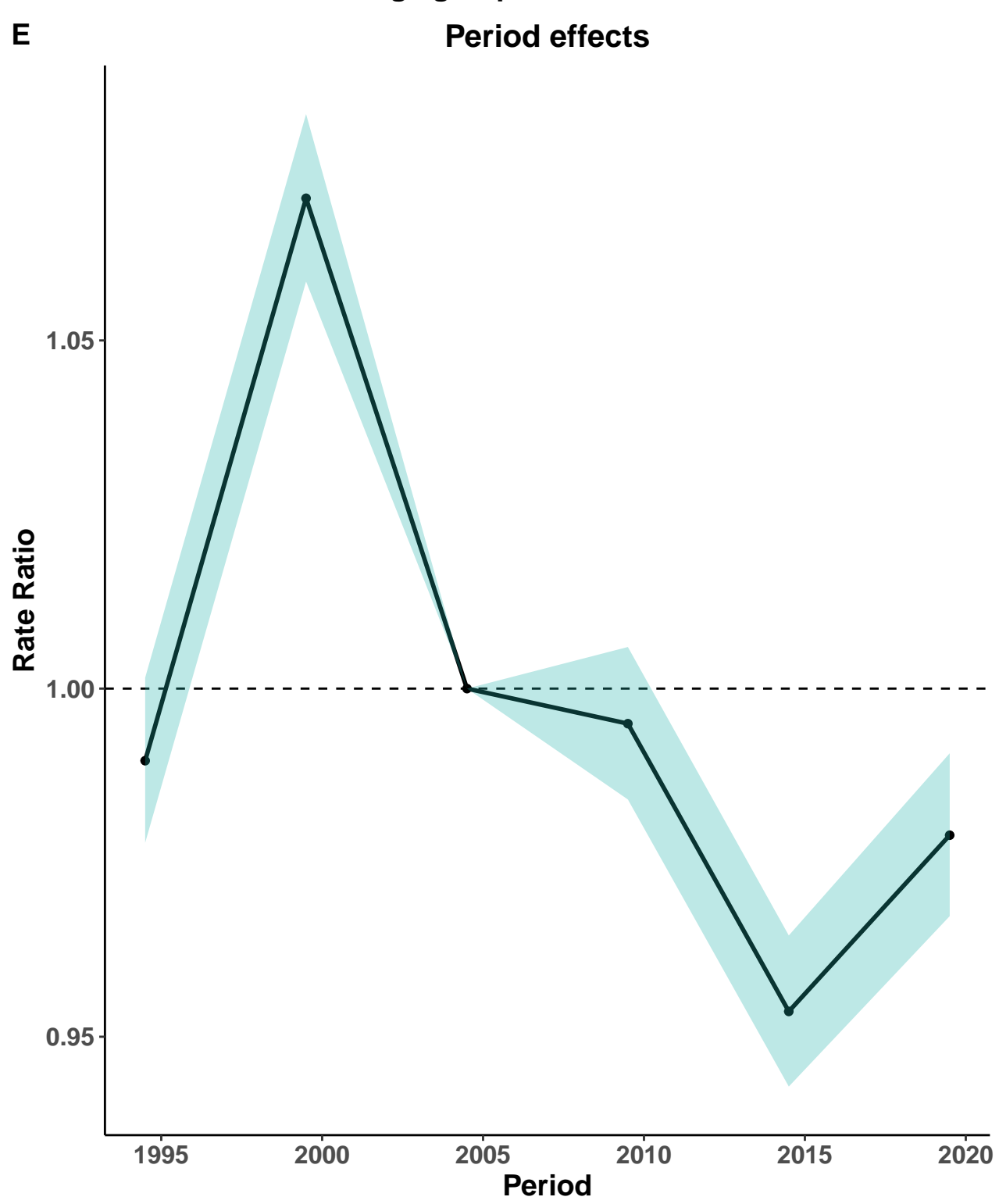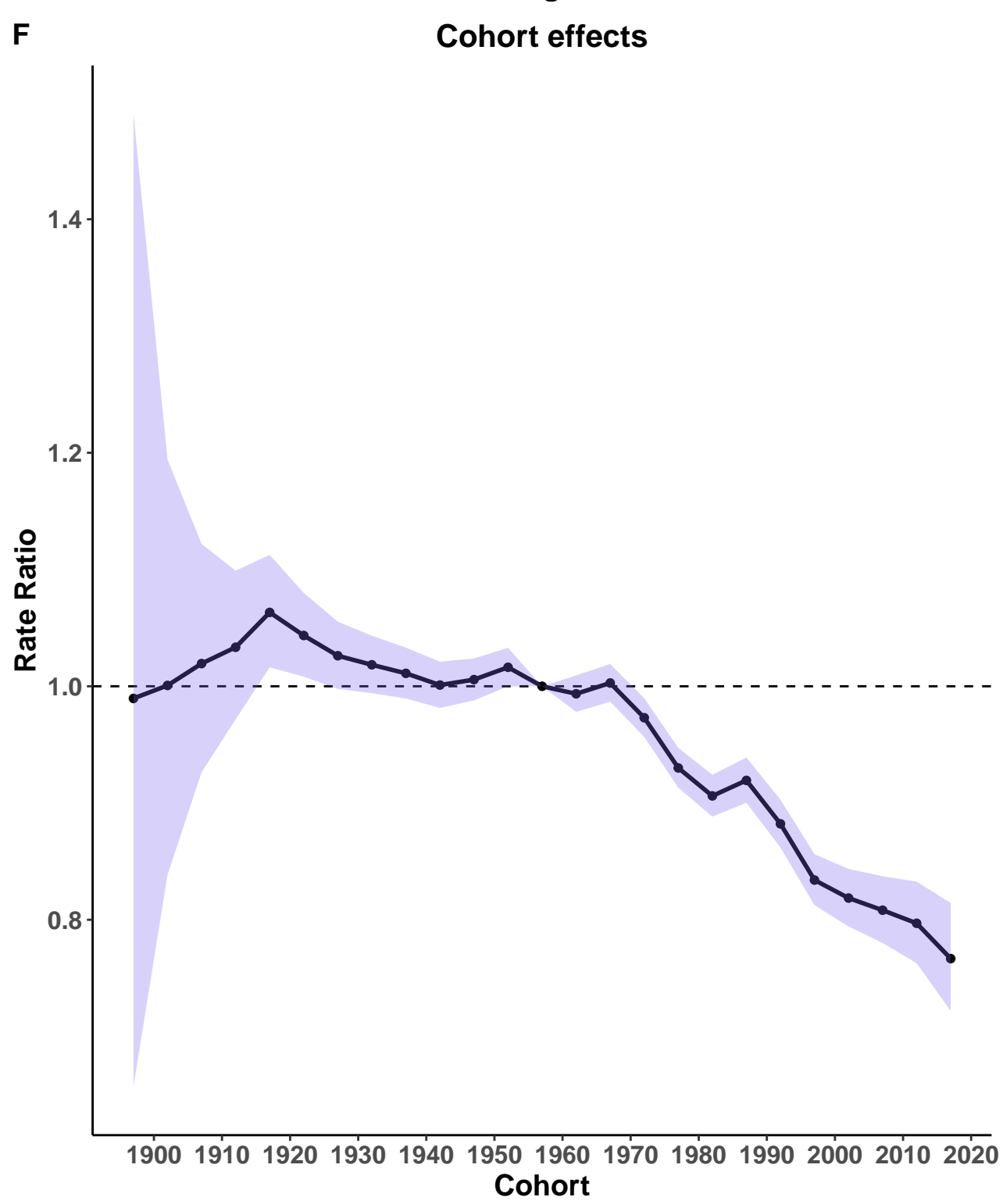

Supplement: Supplementary Figure 2 — Age–Period–Cohort analysis of age-standardized rates for Incidence of EMBID. Panels show (A) cohort-specific rates by age group, (B) age-specific rates by period, (C) net drift and local drifts, (D) age effects, (E) period effects, and (F) cohort effects. [file DataSheet2.pdf]

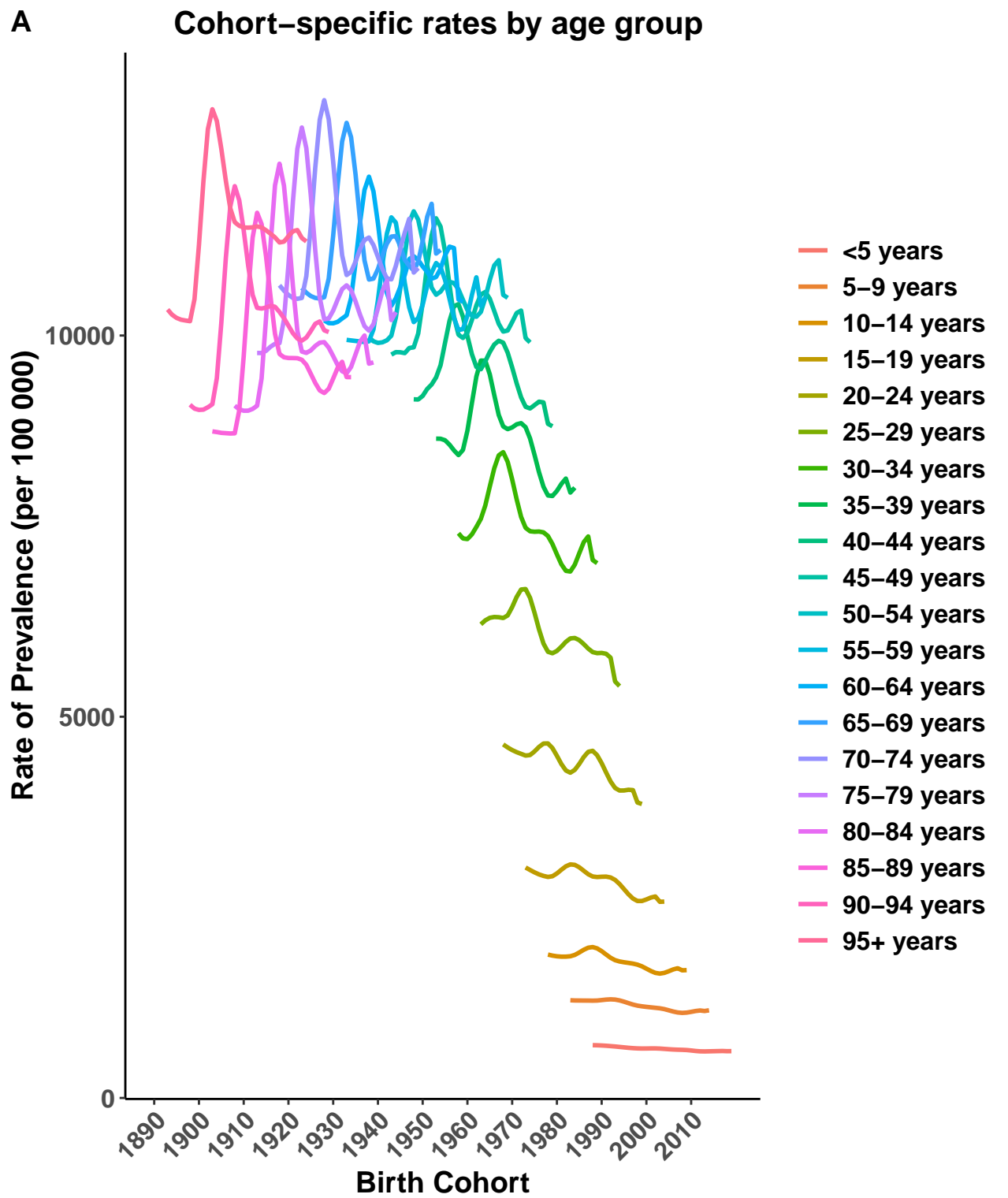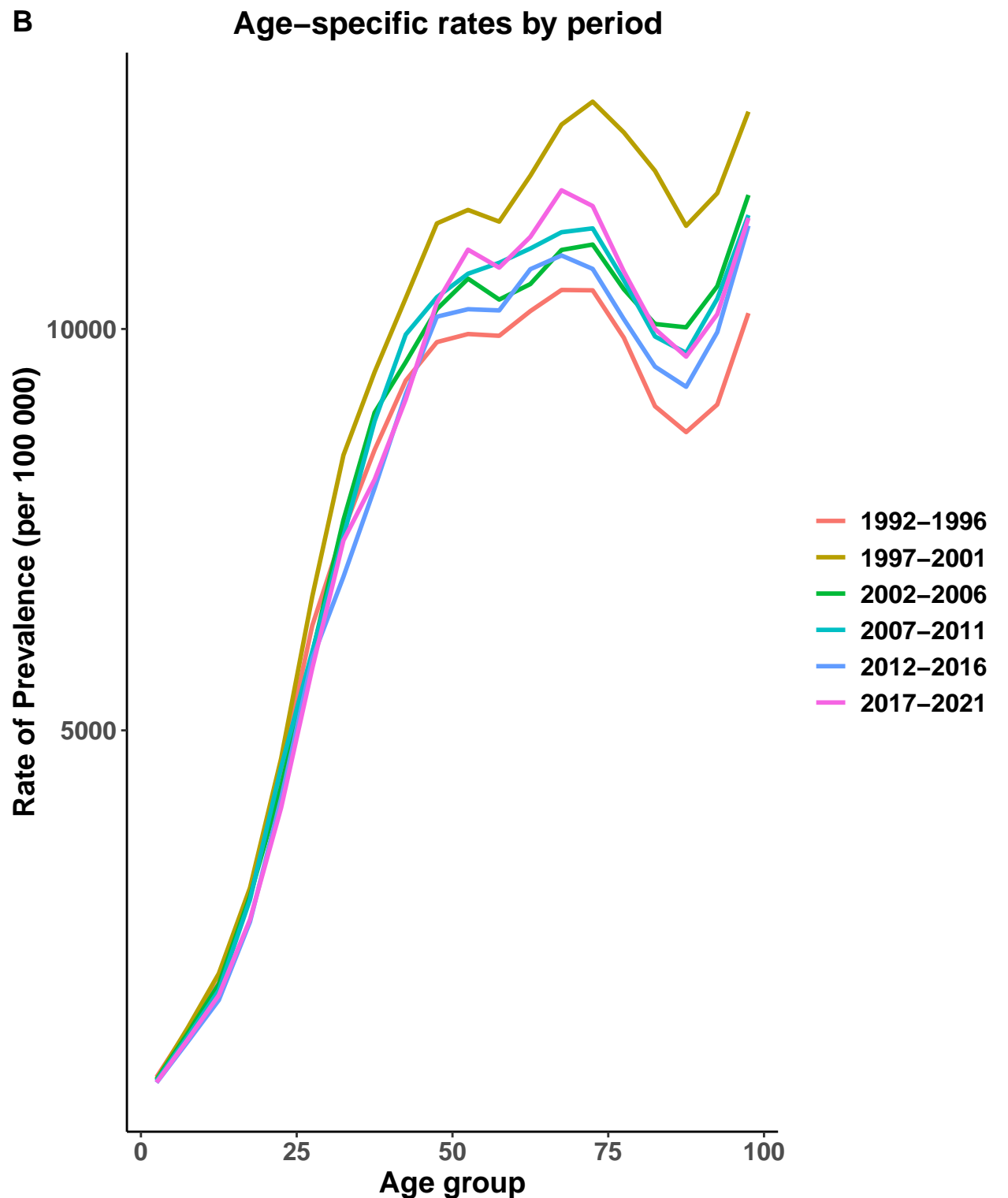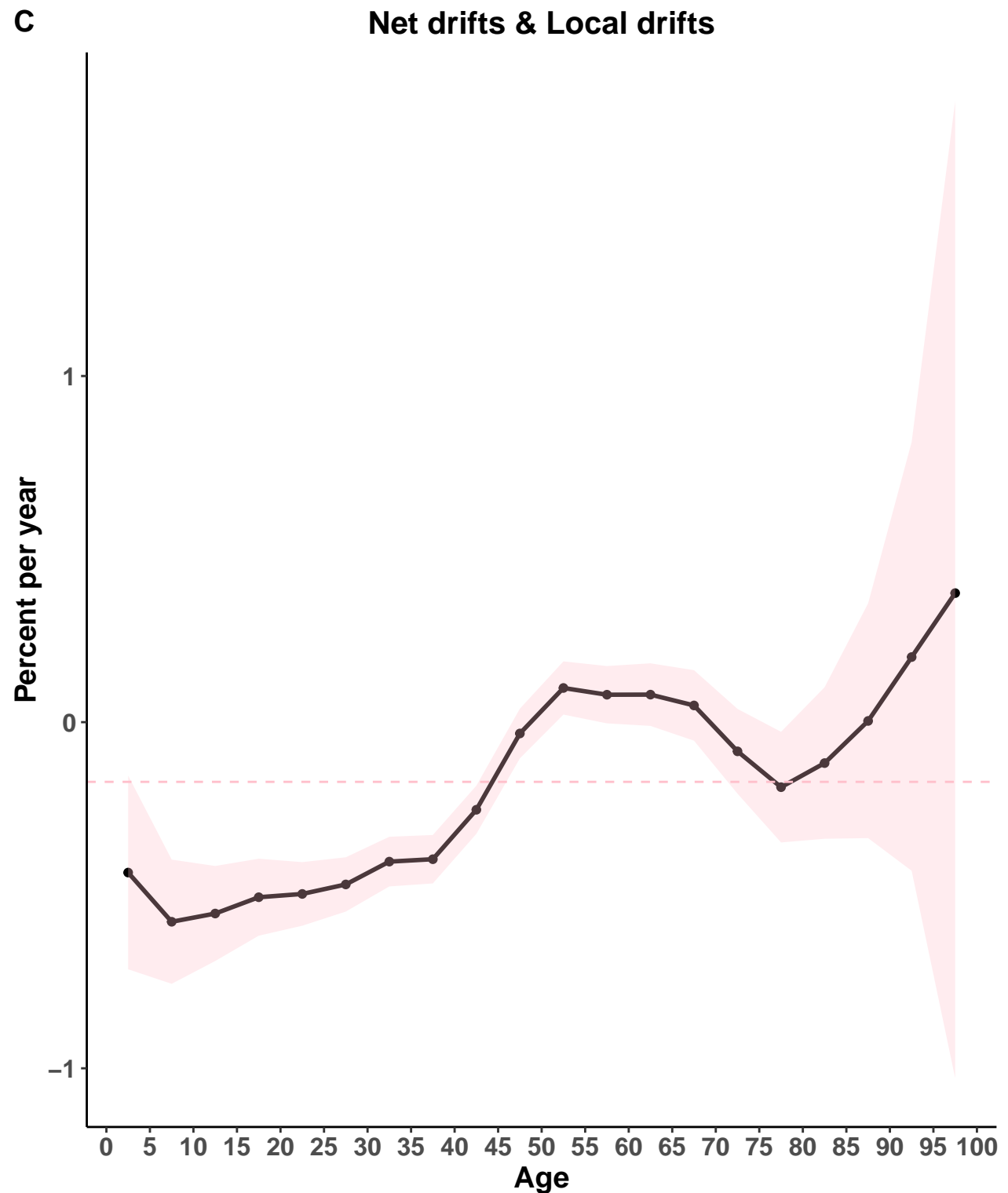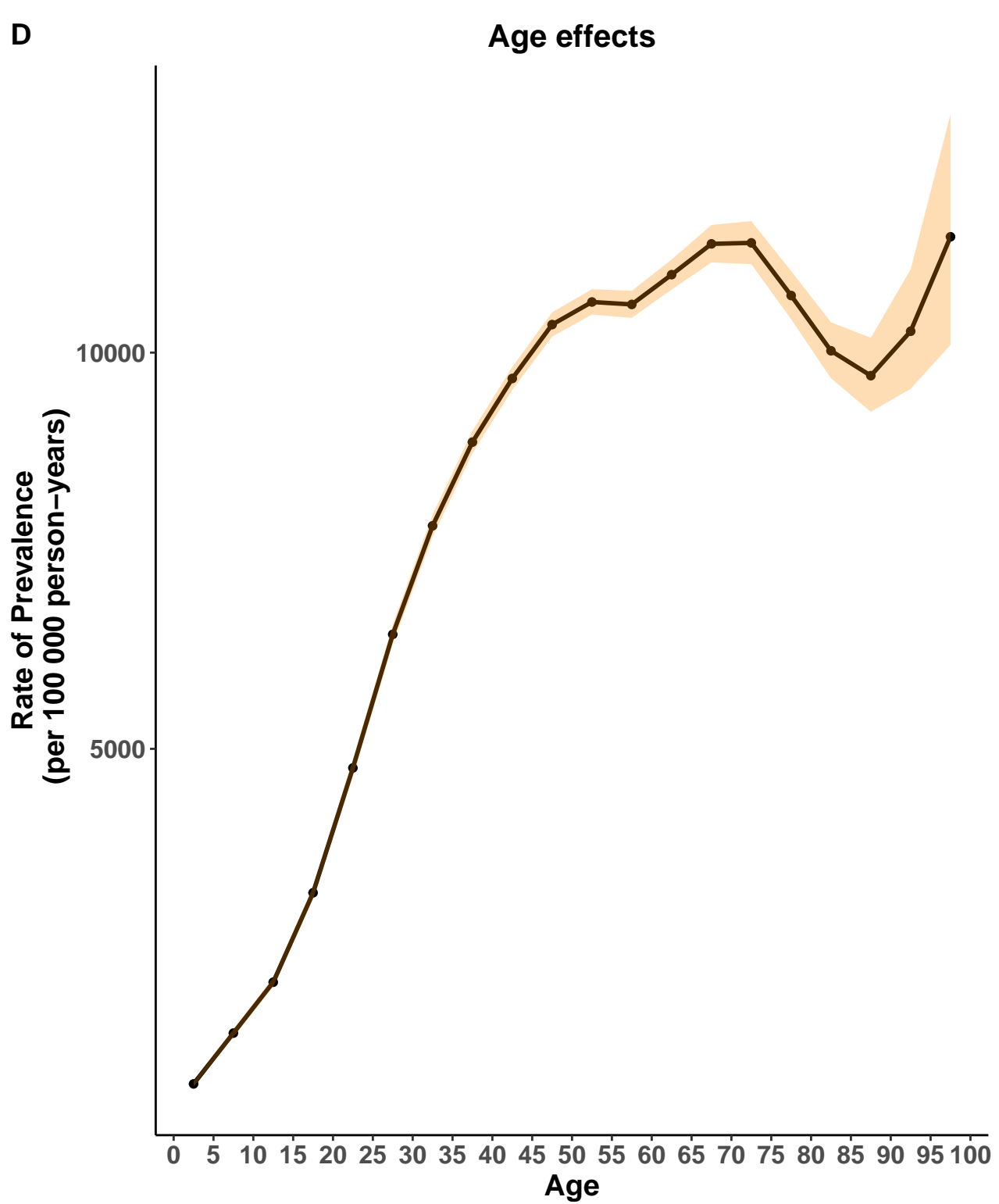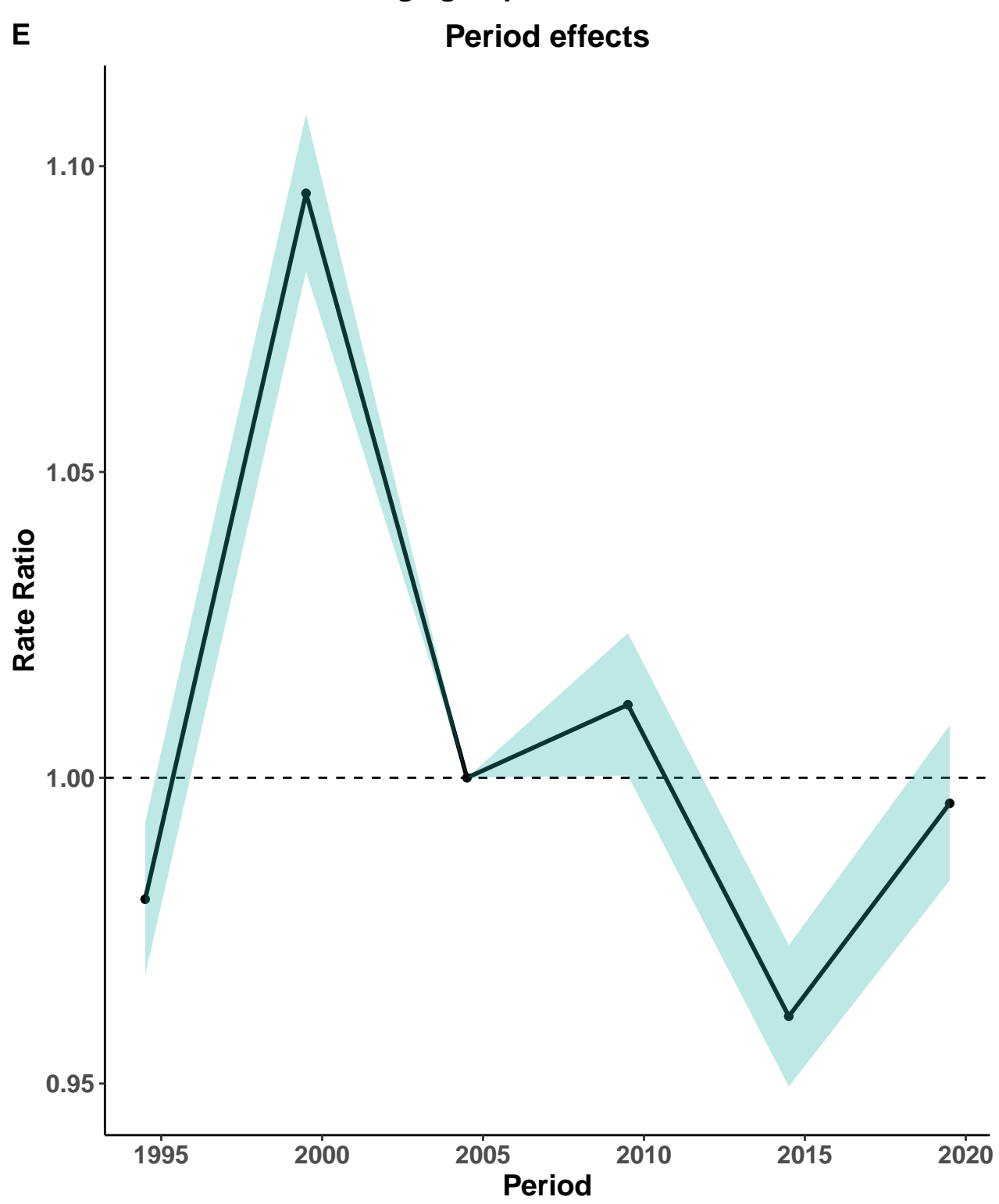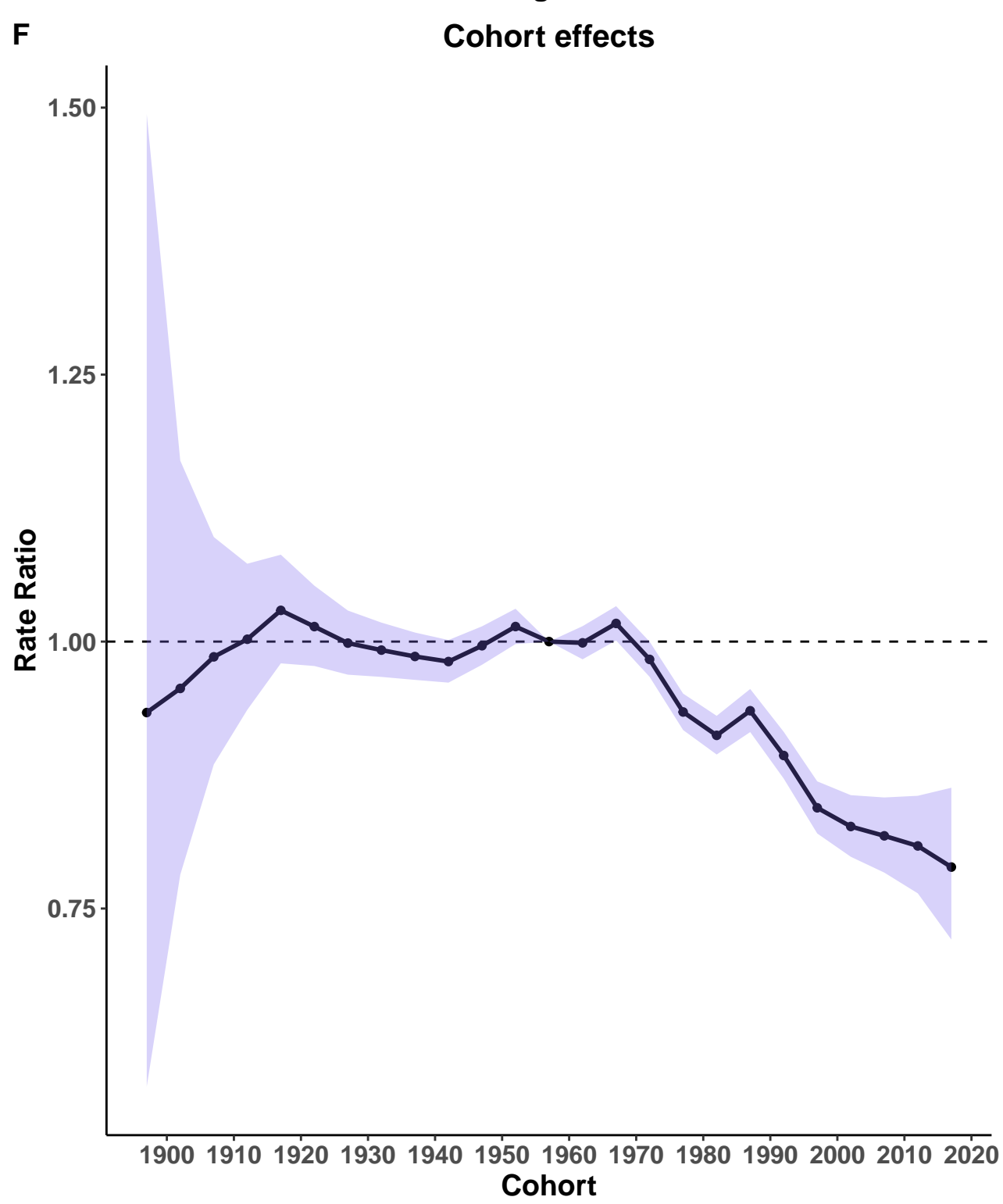

Supplement: Supplementary Figure 3 — Age–Period–Cohort analysis of age-standardized rates for Prevalence of EMBID. Panels show (A) cohort-specific rates by age group, (B) age-specific rates by period, (C) net drift and local drifts, (D) age effects, (E) period effects, and (F) cohort effects. [file DataSheet3.pdf]
