# Supplementary material for: Global, regional, and national burden of endocrine, metabolic, blood, and immune disorders from 1990 to 2021, and projections to 2050: a systematic analysis of the global burden of disease study
Source: Front Endocrinol (Lausanne). 2025 Jul 25;16:1631123. doi: 10.3389/fendo.2025.1631123 (PMC12331491; doi:10.3389/fendo.2025.1631123)
Supplement: Supplementary file 5 [file Table2.docx]

|  | Deaths,95%UI | | DALYs,95%UI | | Incidence,95%UI | | Precalence,95%UI | |
| --- | --- | --- | --- | --- | --- | --- | --- | --- |
|  | 1990 | 2021 | 1990 | 2021 | 1990 | 2021 | 1990 | 2021 |
| **Global** | 78445 (67529, 89083) | 175902 (154306, 190755) | 8184181 (6161723, 10871700) | 12862748 (9943683, 16975278) | 49090191 (39522648, 61098831) | 79473878 (63335600, 98633108) | 284951512 (224216454, 354871071) | 475781303 (381231005, 591192459) |
| Sex |  |  |  |  |  |  |  |  |
| Male | 36414 (31151, 38718) | 85076 (74139, 91589) | 3198478 (2507629, 4046474) | 5050158 (4049189, 6306811) | 15793781 (12562339, 19806007) | 25409383 (20150808, 31921098) | 90570902 (70175565, 114185727) | 151429583 (119600006, 189793639) |
| Female | 42031 (33108, 51680) | 90826 (76371, 100633) | 4985703 (3531843, 6865433) | 7812590 (5796338, 10717853) | 33296409 (26901203, 41299519) | 54064495 (43234151, 66722045) | 194380610 (154040889, 240494418) | 324351719 (260996846, 401238467) |
| **SDI** |  |  |  |  |  |  |  |  |
| High SDI | 27252 (25866, 28045) | 73113 (65534, 77296) | 1509445 (1257381, 1862913) | 2636931 (2279660, 3147375) | 6385361 (5146710, 7904995) | 9853456 (7954190, 12312010) | 41744696 (33363038, 51698991) | 63170213 (51341558, 76435596) |
| High-middle SDI | 14704 (13265, 18237) | 26264 (23191, 29767) | 1798483 (1334742, 2478537) | 2429955 (1786641, 3363177) | 11599732 (9324202, 14451857) | 17234561 (13622814, 21613570) | 70854841 (55744817, 88724079) | 110219720 (87166955, 138044966) |
| Middle SDI | 22079 (16935, 25435) | 46918 (37247, 52018) | 2926720 (2120446, 3959515) | 4356422 (3268493, 5923668) | 18923721 (15092119, 23697361) | 30053507 (23658714, 37346372) | 108693617 (84801375, 136660094) | 180687223 (145129188, 225425196) |
| Low-middle SDI | 8975 (6738, 11914) | 19386 (15515, 22775) | 1326531 (962788, 1801931) | 2252521 (1658420, 3002321) | 8731283 (6976171, 10876143) | 14995924 (12008578, 18663000) | 46058725 (36061692, 57879497) | 83111913 (65272783, 104111016) |
| Low SDI | 5309 (3307, 8175) | 10031 (7026, 12562) | 612619 (418631, 897564) | 1173762 (858479, 1534100) | 3402386 (2729820, 4242410) | 7266360 (5810500, 9143938) | 17328492 (13626238, 21716784) | 38183135 (30047075, 48039567) |
| **Age** |  |  |  |  |  |  |  |  |
| <5 years | 17080 (10858, 24114) | 10551 (7635, 12813) | 1599340 (1037267, 2240925) | 1011487 (767601, 1218292) | 1615173 (1082534, 2258196) | 1489859 (986381, 2115471) | 4291869 (2877975, 6006237) | 4039476 (2698305, 5680366) |
| 5-9 years | 1923 (1485, 2369) | 1493 (1197, 1732) | 298162 (223702, 392878) | 268718 (199700, 376863) | 1732034 (1006969, 2682132) | 1836158 (1057906, 2845581) | 7475480 (4689735, 11253349) | 7896400 (4916538, 11901307) |
| 10-14 years | 1472 (1220, 1718) | 1490 (1217, 1710) | 288851 (200013, 418086) | 307777 (215818, 452270) | 2323584 (1261073, 4065494) | 2579376 (1398265, 4536618) | 10074222 (6283807, 16418887) | 11176057 (6964643, 18272743) |
| 15-19 years | 1618 (1323, 1852) | 1698 (1371, 1903) | 373838 (243606, 598263) | 384169 (254245, 619253) | 3317766 (1846586, 5739574) | 3399008 (1889656, 5913872) | 15700577 (9016819, 25751934) | 16071332 (9107761, 26355060) |
| 20-24 years | 1736 (1408, 1985) | 2056 (1701, 2252) | 485043 (303507, 789333) | 505107 (316821, 814652) | 4232772 (2303700, 6805600) | 4308808 (2325635, 6984929) | 22816594 (12700722, 36609062) | 23022915 (12706050, 37532132) |
| 25-29 years | 1921 (1561, 2179) | 2748 (2282, 3024) | 556891 (331273, 909052) | 670715 (419207, 1066512) | 4491045 (2652491, 7186022) | 5267705 (3084592, 8398150) | 27497092 (15672277, 41664141) | 31779494 (17798010, 48326102) |
| 30-34 years | 2159 (1815, 2395) | 3865 (3150, 4225) | 579081 (351812, 963666) | 885311 (556508, 1452082) | 4317113 (2299723, 6952639) | 6441315 (3440337, 10413471) | 28544661 (17137555, 48668557) | 42397302 (25484819, 72387954) |
| 35-39 years | 2466 (2082, 2766) | 4777 (3942, 5235) | 619137 (365873, 997679) | 960673 (603871, 1504399) | 4471103 (2489179, 7302164) | 6621954 (3692628, 10784622) | 30460055 (16829282, 47026074) | 44892497 (24786523, 69459468) |
| 40-44 years | 2543 (2208, 2830) | 5618 (4731, 6121) | 545260 (330710, 873080) | 966979 (615929, 1502015) | 3920363 (2199951, 6670640) | 6537698 (3677088, 11126619) | 26251311 (15917308, 41262537) | 44067993 (26598307, 69417208) |
| 45-49 years | 2723 (2328, 2990) | 6969 (5957, 7594) | 480818 (303174, 753238) | 1036328 (684940, 1579816) | 3436642 (1893116, 5381248) | 6926600 (3799875, 10884985) | 22637835 (13840628, 35097187) | 46922117 (28648796, 71901468) |
| 50-54 years | 3428 (2935, 3823) | 8940 (7704, 9791) | 472320 (310948, 732546) | 1072127 (730528, 1626134) | 3221000 (1855678, 5053756) | 6813396 (3900906, 10745049) | 21208950 (12801188, 33046958) | 46688741 (27764274, 72272509) |
| 55-59 years | 4049 (3568, 4544) | 11167 (9909, 12054) | 436003 (294242, 650510) | 1035181 (726244, 1515724) | 2942770 (1703878, 4701641) | 6409734 (3694070, 10275269) | 18414532 (11393805, 29467767) | 41550297 (25877856, 66936910) |
| 60-64 years | 4975 (4393, 5519) | 13228 (11898, 14437) | 412997 (290737, 621215) | 921880 (683400, 1337165) | 2892567 (1693855, 4831119) | 5787380 (3386131, 9666818) | 16379275 (10017038, 25491215) | 33419382 (20674046, 51698047) |
| 65-69 years | 5798 (5223, 6523) | 15436 (13727, 16748) | 356111 (259685, 498008) | 871344 (653408, 1183289) | 2456594 (1394543, 3966745) | 5660678 (3214193, 9010601) | 13132340 (8539134, 19788625) | 30677783 (20138308, 45871606) |
| 70-74 years | 5684 (5135, 6505) | 16780 (15155, 18164) | 260494 (191147, 348328) | 697546 (531552, 915250) | 1656957 (984590, 2643838) | 4061336 (2413021, 6509372) | 9024044 (5285749, 14066932) | 22404504 (13226919, 34553584) |
| 75-79 years | 6561 (6006, 7223) | 16681 (14848, 17974) | 203907 (160533, 267634) | 487966 (390497, 630333) | 1096175 (673358, 1717218) | 2455805 (1509521, 3821742) | 6014693 (3929916, 9073230) | 13593701 (8846105, 20287503) |
| 80-84 years | 5653 (5007, 6354) | 16951 (14562, 18555) | 123439 (100638, 155822) | 347805 (287545, 428342) | 606671 (385355, 862536) | 1578873 (1011389, 2238265) | 3212013 (2132607, 4596430) | 8449435 (5659984, 12160359) |
| 85-89 years | 4134 (3586, 4566) | 16529 (13544, 18256) | 62643 (51905, 75134) | 232454 (192300, 276774) | 259582 (165861, 375525) | 830524 (530233, 1201468) | 1321081 (885329, 1869991) | 4322543 (2898735, 6138388) |
| 90-94 years | 1897 (1568, 2099) | 12358 (9501, 13870) | 22984 (19256, 27379) | 136109 (110531, 160350) | 78969 (50731, 122692) | 349377 (224764, 544841) | 389608 (259530, 608089) | 1797051 (1204364, 2777319) |
| 95+ years | 626 (483, 712) | 6566 (4691, 7546) | 6861 (5575, 8171) | 63071 (47804, 72736) | 21311 (10766, 36583) | 118294 (59101, 201364) | 105279 (65154, 165925) | 612283 (375333, 956991) |
| **21 Regions** |  |  |  |  |  |  |  |  |
| Andean Latin America | 1351 (858, 1576) | 1517 (1219, 2336) | 101603 (65897, 123719) | 97829 (75141, 132376) | 238075 (189086, 296429) | 508507 (404748, 627067) | 1390452 (1098147, 1739818) | 3055345 (2475734, 3792661) |
| Australasia | 606 (573, 634) | 2002 (1768, 2143) | 29681 (25728, 35047) | 63449 (55987, 72894) | 104413 (82738, 130854) | 194986 (156038, 240717) | 645197 (515104, 796503) | 1182548 (965882, 1442201) |
| Caribbean | 1852 (1493, 2383) | 2719 (2260, 3429) | 132169 (98903, 181956) | 161068 (124566, 212242) | 447099 (364844, 551053) | 715860 (581091, 868221) | 2322770 (1864211, 2873077) | 3854491 (3157915, 4624745) |
| Central Asia | 411 (375, 483) | 1051 (911, 1195) | 72569 (54870, 96869) | 121147 (92900, 158855) | 440534 (352644, 555052) | 640764 (505991, 798845) | 2547994 (1973810, 3181211) | 3953621 (3133490, 4912066) |
| Central Europe | 1912 (1820, 1987) | 2084 (1911, 2256) | 183754 (147422, 235733) | 165435 (127851, 217279) | 984536 (792253, 1233226) | 1072395 (851338, 1341866) | 5710486 (4601739, 7055089) | 6229119 (5127334, 7602092) |
| Central Latin America | 3146 (3045, 3253) | 8914 (8052, 9762) | 252061 (213008, 306025) | 502124 (418359, 611069) | 1182573 (945825, 1482870) | 2561343 (2056095, 3162929) | 6450639 (5081353, 8121782) | 14666393 (11915393, 18013867) |
| Central Sub-Saharan Africa | 942 (360, 1795) | 1907 (841, 2917) | 89512 (45370, 161021) | 173689 (101873, 251125) | 295511 (235494, 367056) | 765560 (601969, 967810) | 1637098 (1275112, 2057150) | 4285738 (3360964, 5377348) |
| East Asia | 14037 (10487, 17318) | 20066 (13856, 24946) | 2458018 (1693247, 3450025) | 3074228 (2091491, 4506307) | 17246868 (13660169, 21608403) | 25402773 (19784226, 31967862) | 107437586 (84218752, 136432409) | 167360752 (132065750, 212047807) |
| Eastern Europe | 1569 (1541, 1597) | 2840 (2646, 3036) | 291558 (215431, 398419) | 311388 (234797, 416020) | 2417661 (1926815, 3018615) | 2451833 (1953782, 3085116) | 12634182 (10258917, 15688683) | 12895437 (10497587, 15657581) |
| Eastern Sub-Saharan Africa | 2080 (1475, 2811) | 4229 (2941, 5609) | 220216 (160799, 295148) | 432098 (313872, 568822) | 1272773 (1024912, 1592748) | 2714255 (2156133, 3419732) | 6429601 (5081701, 8023814) | 14215148 (11089676, 17922729) |
| High-income Asia Pacific | 2560 (2372, 2799) | 5728 (4613, 6403) | 146469 (123693, 177925) | 178001 (148561, 216243) | 639970 (511118, 793141) | 862092 (689714, 1080909) | 3550993 (2910542, 4334360) | 4732776 (3920522, 5672866) |
| High-income North America | 9992 (9489, 10305) | 44049 (39654, 46416) | 553232 (463779, 682163) | 1349207 (1216517, 1531267) | 2446801 (1943523, 3039990) | 3854087 (3101263, 4806098) | 15400460 (12056718, 19483359) | 23392477 (19154657, 28540043) |
| North Africa and Middle East | 6178 (4279, 11452) | 12154 (9800, 15793) | 607395 (428170, 1019346) | 997540 (780484, 1261009) | 2353041 (1893815, 2933243) | 4872286 (3897097, 6050559) | 12478680 (9822823, 15701849) | 27180001 (21602013, 34203523) |
| Oceania | 136 (85, 196) | 350 (224, 476) | 13006 (8820, 18082) | 31699 (21993, 42478) | 66523 (52623, 84073) | 156693 (124912, 196300) | 417999 (325306, 529083) | 1040506 (806009, 1306681) |
| South Asia | 3957 (2657, 5316) | 7248 (5355, 8374) | 953976 (657576, 1328110) | 1610314 (1132209, 2293867) | 7843658 (6249882, 9835243) | 13499106 (10733746, 16883396) | 39167302 (30594107, 49665058) | 70700282 (55566235, 89834628) |
| Southeast Asia | 4311 (3074, 5377) | 9406 (7111, 11651) | 722101 (490449, 1021723) | 1235622 (871735, 1772979) | 5747241 (4590428, 7181174) | 9585746 (7597263, 11834996) | 32834352 (25808108, 40918330) | 58873633 (46787115, 74089443) |
| Southern Latin America | 2474 (2336, 2586) | 2081 (1924, 2198) | 113187 (103024, 126323) | 99427 (84155, 121027) | 261120 (210677, 322578) | 405235 (325441, 499631) | 1644026 (1345393, 2006704) | 2627543 (2151359, 3178040) |
| Southern Sub-Saharan Africa | 1767 (1462, 2405) | 6185 (3896, 7340) | 116647 (94710, 149869) | 311274 (218686, 372508) | 374337 (301639, 466234) | 730467 (587107, 896316) | 1986283 (1576120, 2466666) | 4001971 (3246069, 4919579) |
| Tropical Latin America | 2154 (2042, 2294) | 11214 (10409, 11760) | 194624 (156320, 248541) | 534200 (460849, 641301) | 1141028 (909136, 1421246) | 2385614 (1905303, 2968660) | 5920518 (4643901, 7481275) | 12749764 (10468372, 15487667) |
| Western Europe | 14615 (13796, 15051) | 25205 (22462, 26930) | 704081 (597735, 857183) | 922098 (785065, 1114517) | 2386056 (1930374, 2962917) | 3186319 (2571256, 3926443) | 18186623 (14835219, 21999473) | 23559412 (19526219, 28089561) |
| Western Sub-Saharan Africa | 2397 (1333, 3327) | 4951 (3438, 6131) | 228321 (150050, 314384) | 490911 (363356, 649348) | 1200372 (960874, 1496403) | 2907960 (2312873, 3643423) | 6158270 (4855974, 7730211) | 15224344 (11890610, 19168090) |

Supply Table1 Global and Regional Mortality, DALYs, Incidence, and Prevalence cases of EMBID by Sex, Age, and SDI Regions
